# Integrating Omics and Gene Editing Tools for Rapid Improvement of Traditional Food Plants for Diversified and Sustainable Food Security

**DOI:** 10.3390/ijms22158093

**Published:** 2021-07-28

**Authors:** Ajay Kumar, Thattantavide Anju, Sushil Kumar, Sushil Satish Chhapekar, Sajana Sreedharan, Sonam Singh, Su Ryun Choi, Nirala Ramchiary, Yong Pyo Lim

**Affiliations:** 1 Department of Plant Science, Central University of Kerala, Kasaragod 671316, Kerala, India; anjut609@gmail.com (T.A.); sajanasreedharan@gmail.com (S.S.); 2 Department of Botany, Govt. Degree College, Kishtwar 182204, Jammu and Kashmir, India; sushilthakur863@gmail.com; 3 Molecular Genetics & Genomics Laboratory, Department of Horticulture, Chungnam National University, Daejeon 34134, Korea; sushilchhapekar@gmail.com (S.S.C.); sonamsingh688@gmail.com (S.S.); srchoi@cnu.ac.kr (S.R.C.); 4 School of Life Sciences, Jawaharlal Nehru University, New Delhi 110067, Delhi, India

**Keywords:** traditional food plants, climate change, food security, omics, translational genomics, gene editing, CRISPR/Cas, COVID-19

## Abstract

Indigenous communities across the globe, especially in rural areas, consume locally available plants known as Traditional Food Plants (TFPs) for their nutritional and health-related needs. Recent research shows that many TFPs are highly nutritious as they contain health beneficial metabolites, vitamins, mineral elements and other nutrients. Excessive reliance on the mainstream staple crops has its own disadvantages. Traditional food plants are nowadays considered important crops of the future and can act as supplementary foods for the burgeoning global population. They can also act as emergency foods in situations such as COVID-19 and in times of other pandemics. The current situation necessitates locally available alternative nutritious TFPs for sustainable food production. To increase the cultivation or improve the traits in TFPs, it is essential to understand the molecular basis of the genes that regulate some important traits such as nutritional components and resilience to biotic and abiotic stresses. The integrated use of modern omics and gene editing technologies provide great opportunities to better understand the genetic and molecular basis of superior nutrient content, climate-resilient traits and adaptation to local agroclimatic zones. Recently, realizing the importance and benefits of TFPs, scientists have shown interest in the prospection and sequencing of TFPs for their improvements, cultivation and mainstreaming. Integrated omics such as genomics, transcriptomics, proteomics, metabolomics and ionomics are successfully used in plants and have provided a comprehensive understanding of gene-protein-metabolite networks. Combined use of omics and editing tools has led to successful editing of beneficial traits in several TFPs. This suggests that there is ample scope for improvement of TFPs for sustainable food production. In this article, we highlight the importance, scope and progress towards improvement of TFPs for valuable traits by integrated use of omics and gene editing techniques.

## 1. Introduction

As per Food and Agriculture Organization (FAO) estimates, the global population is expected to reach nine billion by 2050 and the world will have to produce 50% more food than we produce today to feed the burgeoning population [1]. However, increasing the food production of the currently available crops on available land is a challenging task [2]. This challenge is further limited by several factors such as excessive reliance on a limited number of industrialized crops, decreasing land for agriculture and global climate change [2]. Several factors such as desertification and conversion of agricultural lands for non-agricultural activities also pose a major threat to the global food-producing systems [3]. State of the World’s Plants Report 2016 estimated the existence of more than 391,000 species of vascular plants on this planet [4]. This report further estimated that approximately 30,000 species have at least one documented use and more than 5000 of them provide food to humans [5]. It is reported that nearly 2500 species of plants belonging to more than 160 families have undergone domestication throughout the world [6]. Surprisingly, despite having a huge diversity of vascular food plants, the world relies on only a limited number of approximately 15 major crops for 70 percent of food and nutritional requirements that were domesticated by our ancestors more than 10,000 years ago [7,8]. Of the 15 major crops, more than 50 percent of the calories come from five cereal crops, namely wheat, rice, millet, sorghum and maize [7,9]. Excessive reliance on a limited number of mainstream domesticated crops for nutritional requirements has been flagged as an important issue in the global fight against food insecurity and in ensuring global food security to achieve zero hunger by 2030 as envisaged in the Agenda 2030 of Sustainable Development Goals [10]. Furthermore, the current widespread cultivation of uniform domesticated varieties carries huge risks of crop failures and significant reduction in yield as they are more vulnerable to biotic (pathogen and pests) and abiotic stresses (due to global climate change) [11]. It has been estimated that the rise in global mean temperatures may result in a reduction in significant yields of several crops currently in use such as wheat, rice, maize and soybean [12,13]. However, the effect of global climate change is perceived differently by different varieties/crops, and in different regions of the world [14,15]. It further necessitates the identification of the local species/varieties that are used in different agro-climatic regions of the world [16]. Therefore, identification of new crops and varieties with superior nutrition content suitable to the local agro-climatic zones is an important agenda for plant scientists [16,17]. 

A number of recent studies have pointed towards the exploration and exploitation of traditionally used food crops (TFPs) for nutritional food security and their mainstreaming [18,19,20]. TFPs can act as supplementary diets and also as emergency foods in times of pandemics or when the global supply chains are disrupted due to man-made or natural disasters. A traditional food crop is an indigenous crop species that is native to a particular region of the world or was introduced from another place long ago and due to its use for generations, it has become a part of the culture of that particular community or region [21,22]. Several local indigenous communities of the world still use and rely on such traditional crops which were in use for centuries but are currently neglected, underutilized, restricted to particular geographical locations and are not in mainstream use [23]. Nevertheless, recent years have seen increased preference of consumers towards these ancient traditional varieties and there is an increased focus on the reintroduction and mainstreaming of such traditionally used ancient food crop varieties [24,25,26,27]. Considering the nutritional, economic and agricultural importance of TFPs and their use as future climate-resilient crops, it is important to explore the application of the modern omics technologies for dissection of molecular mechanisms governing those traits [28]. Furthermore, the extensive exploitation of genetic diversity is required to address the vulnerability of crop plants due to the narrow genetic base [29]. Modern technologies can be used to characterize the crop germplasm collections to be used for better and sustainable food production and supply; for example, Milner et al. [30] and Langridge and Waugh [31] evaluated more than 20,000 wild and domesticated barley genotypes with the aid of genotyping and informatics technologies and demonstrated the scope of exploitation of genetic resources in crop improvement [30,31].

This review article discusses the potential use of various omics technologies for understanding the genetic makeup, proteomes, metabolomes, ionomes and nutritional composition of TFPs. This review also provides details about the use of available genomics information from model crops and its potential in translational research of TFPs. We further discussed a detailed futuristic outline of integrated use of omics and gene editing technologies for rapid improvement/domestication of TFPs.

## 2. Importance of Traditional Food Plants

### 2.1. Diversity of Traditional Food Plants across the Globe

Incidences of crop failures triggered by climate change and pathogens are expected to rise in the future [32]. We have already experienced such crop failures in the past; for example, over-dependence on the potato and the attack of *Phytophthora infestans* resulted in the Irish famine which led to starvation, widespread deaths and emigrations to the other parts of the world [33]. Southern leaf blight of corn in the United States is another example of the risks of a single crop or one type of crop carrying pathogens [34]. There are several other examples of major crop failures from across the world, indicating the potential risks inherent to the cultivation of less diversified and uniform crops [35,36]. The uniform varieties are most likely to be destroyed simultaneously with the evolution of resistant pathogens or with climate change unless region-specific strategies and preventive measures are in place [37]. This leads to widespread hunger, malnutrition, migration and may even lead to civil wars [11,38]. Therefore, the existence of diversity in food plants is crucial and required for the breeding of improved varieties for desirable traits such as stress resistance and nutritional superiority [39,40,41]. It is also desirable to ensure healthy, sustainable food security, to reduce the impacts of diseases and climate change and to improve the stability of food production [42,43]. Minor TFPs have so far largely been ignored and much attention is not given to them for their role in sustainable food security because of certain undesirable characteristics and their restricted geographical availability [44]. However, recent years have seen an increased interest in the revival of the traditional plants and the food systems that are based on the TFPs [43,44,45,46]. Efforts across the globe are ongoing to diversify the currently cultivated basket of food crops, to provide more options to the farmers to grow crops and to the consumers for diversifying their food menu [47]. Large amounts of fragmented ethnobotanical data on TFPs are available from various countries [48]. Several studies have performed their nutritional and stress-related analysis and results from these studies suggest the potential roles of TFP diversity in fighting against the hidden hunger of the world by ensuring global food security [49]. 

The diversification of nutritionally rich and stress-resilient traditional, orphan and underutilized crops can help to achieve the goal of a zero-hunger world as envisaged in the United Nations Sustainable Development Goals (SDGs), which specifically propose to end hunger, achieve food security, improve nutrition and promote sustainable agriculture globally by 2030 [50,51]. However, extensive research is needed on TFPs to integrate them into global food systems [51]. It is necessary to understand consumption barriers as well as production constraints [52]. Although TFPs are very important for food security [53], many of them produce relatively lesser yields due to the lack of selection of improved traits. They are also not cultivated on a large scale because of unfavorable policies for their promotion [54]. However, several initiatives have recently been taken that are focused on the promotion of TFPs and improvement of their traits with the aid of genetic and genomic tools [54]. For example, African Orphan Crops Consortium (AOCC) is involved in the sequencing of 101 orphan crops and their integration into African food production-consumption systems [55]. The AOCC is a global partnership dedicated to the genome-enabled advancement of 101 African orphan crops that have superior nutrient and adaptive characteristics [52,56]. The consortium is aimed to elucidate reference genomes of 101 species for exploring genetic diversity. AOCC is an important model that can be adopted in other parts of the world especially to those areas which have rich diversity of TFPs [52]. Similarly, there exists an independent international organization named Crops For the Future (CFF) which aims to promote and facilitate the use of underutilized, neglected and orphan crops and their integration into human diets. The mission of CFF includes increasing the knowledge base of neglected crops, advocating policies related to promotion of neglected crops and spreading awareness about the relevance of neglected crops for rural livelihoods [57]. The Food and Agricultural Organization of the United Nations is also taking initiatives for the promotion of neglected crops by collaborating with agencies such as the International Council for Research in Agroforestry (ICRAF) [58]. Therefore, for attaining sustainability of food production, collective efforts are required to advance the research and development on TFPs [54]. 

### 2.2. Traditional Food Plants Possess Important Nutritional Traits

Experimental evidence suggests that ancient TFPs have certain important nutritional and stress-resilient traits that can be exploited to reduce global hunger and malnutrition under increasing global climate change [59]. TFPs are promising future crops considering their multiple benefits to the farmers, consumers and the environment as well [44,59,60,61]. Traditional crops that are used generation after generation are mostly consumed in a particular region by the local communities for nutritional and therapeutic purposes [62,63]. Several studies have experimentally proven that a number of traditional crops are highly rich in nutritional components, and many of them are resilient to several stresses [19,64]. Some of the examples are the fruit of *Elaeagnus umbellata,* which have ten times higher quantity of lycopene in their fruit than tomato [65], and *Chenopodium quinoa,* which has higher mineral content than maize and barley, including calcium, magnesium, iron, copper, potassium, phosphorus and zinc [66]. Even though they have multiple benefits, the lack of domestication and their cultivation being limited to geographical boundaries hinders their integration into large-scale production systems [23]. Although TFPs possess several important traits, some are also burdened with certain undesirable traits [44]. For example, there are some TFPs with antinutritional components which are harmful when consumed [67]. Therefore, it becomes necessary to remove undesirable traits before they are made available for extensive cultivation and consumption [44]. Prior knowledge of the undesirable traits and the genes governing them is also crucial and we can employ modern gene editing tools to get rid of them. Therefore, rapid domestication of TFPs using gene editing tools is an effective solution for this problem [68]. Redomestication of crops for their wild traits that could be lost due to domestication is another important strategy to access the lost gene pools [69]. 

### 2.3. Traditional Food Plants Show Varying Degrees of Tolerance to Stresses

**Figure 1:**
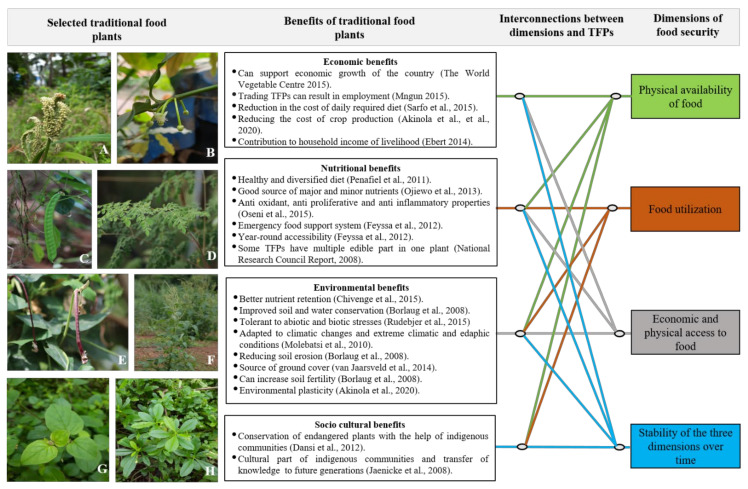
The congruence of traditional food plants with four dimensions of food security [44,58]. Examples of few traditional food plants: (**A**) *Eleusine coracana* (L.) Gaertn., (**B**) *Garcinia madruno* (Kunth) Hammel., (**C**) *Canavalia ensiformis* (L.) DC., (**D**) *Moringa oleifera* Lam., (**E**) *Vigna unguiculata* L. (Walp), (**F**) *Amaranthus palmeri* S. Watson, (**G**) *Boerhavia diffusa* L. and (**H**) *Talinum triangulare* (Jacq.).

FAO has emphasized four important dimensions that determine the food security of a country, region or population viz. enough availability of food, sufficient access to food, food utilization and stability of the first three dimensions [45,70]. Availability of food means enough production of a particular food and its seamless distribution to consumers [42,70]. Sufficient food access indicates economic affordability or freedom to access sufficient food and sufficient allocation of the food resources [71,72]. The third component indicates bio-assimilation of the food that is eaten [70]. The fourth and the last components indicate seamless and sustainable availability of access to and utilization of the food resources [45,71]. The disturbance in the stability of the three dimensions would eventually result in the food insecurity of a region, country or population [73]. Ensuring the food security of a growing population in the future is going to be a challenging task [74]. Various factors affect the components of a healthy and secure food system [45,70]. The production of food is already limited by several factors such as global climate change, biotic and abiotic resources and loss of genetic resources [75]. The sustainable food supply (first component) is disrupted by various factors such as pandemics, wars, natural disasters, droughts, climate change and excessive rainfall [76,77]. Sufficient access to food (second component) is limited by factors such as poverty, food price rises, unemployment, low per capita income and poor market access [78]. If the food is not biologically utilized in the body, it may lead to widespread disease or malnourishment [79]. Therefore, the stability of all three components over time is essential for ensuring sustainable global food security [73]. One of the most important factors that contributes towards the disruption of the stability of the three dimensions of food security is climate change, its associated negative impacts, biotic and abiotic stresses. Such disruptions may result in widespread food insecurity across the globe [78]. A number of studies have reported that climate change and stresses pose serious threats to the growth and reproduction of crop plants and reduce their yields by affecting various processes in the cells [77,80]. Excessive threats of failures that the currently cultivated crops face across the globe necessitate identification of the new climate-resilient crops, and the diversification of the crops [14]. Several studies have also indicated the identification and cultivation of climate-resilient food crops with biotic and abiotic stress tolerance traits [77]. Therefore, there is a larger consensus among various stakeholders about the urgency to identify and promote climate-resilient crops that possess abiotic stress tolerance. Interestingly, a large number of TFPs are adapted to the region of their origin, have huge regional importance to the regional local communities [81], show considerable resilience to climate change and can perform better even under unfavorable environmental conditions [19]. Traditional food plants are more climate-resilient and disease- and pest-resistant, and can survive in harsh environmental conditions [82]. Cultivation of traditional food plants is in congruence with the four important dimensions of food security as defined by FAO [44] (Figure 1). The traditional food systems based on traditional food plants are also resilient and sustainable. The food production, supply and consumption must be sustainable and resilient during times of natural calamities, civil wars or during pandemics when the supply chains are threatened. The current definition of food security therefore also includes sustainability and resilience. The traditional foods and the food systems based on them are sustainable and resilient to such situations. The promotion of climate-resilient, underutilized food crops along with modern crop varieties will be important for stable food production systems, especially under fluctuating environmental conditions [83]. A non-exhaustive list of TFPs with their nutritional and stress-resilient traits is presented in Table 1. 

### 2.4. Traditional Food Plants Ensure Stable and Sustainable Food Security

Stability of food supply, access to food and food utilization over time is important for a healthy food system and ensuring food security [42,45,70]. If concerted efforts are not taken in the immediate future to revive and conserve them, they may disappear from the global food menu [25,213,214]. This will contribute to the loss of genetic diversity and resources important for breeding the nutritionally superior and climate-resilient varieties [215,216,217]. Therefore, it becomes necessary to enhance our focus from the model and select domesticated crops towards less-consumed and neglected traditional crops that hold promising potential in alleviating global hunger and ensuring food security [218]. There is an increasing interest among scientists in the exploitation of TFPs, understanding their genetic basis of important traits and further improvement. However, breeding improved varieties that are nutritionally superior and climate-resilient requires a complete understanding of the genetic and molecular basis of such traits [219]. Recent technological advancements in the high throughput omics approaches provide opportunities to dissect the genetic and molecular basis of nutritional and stress tolerance-related traits. Integration of multi-omics tools such as genomics, transcriptomics, metabolomics, proteomics and ionomics can help us comprehensively investigate the gene–protein–metabolite networks of nutrition, climate resilience and other traits [220]. In an interconnected, interdependent and globalized world, several countries are involved in bilateral and multilateral trades in food and food-related products [221]. Situations such as global pandemics, wars and physical disruptions to logistics can disrupt global food supply chains, resulting in global, regional or local food insecurity endangering a large population [222]. Currently, COVID-19 has threatened multilateral and bilateral trades between nations [223]. The supply of food from one country to another is severely affected [224]. Some countries which are excessively dependent on the import of food grains are the most affected due to COVID-19 [225]. Such pandemic-related disruptions in food security can be averted if foods are locally grown and made available for the local populations [226]. Additionally, the cultivation of local varieties promotes local agriculture and conserves the biodiversity of the local agroecosystems [227]. It has also been argued that consumption of locally grown foods may be advantageous over long-distance foods, as locally harvested foods are almost available in less time to the consumers and their freshness ensures that they are of better nutritional quality [228]. The promotion of TFPs will also promote the role of local communities in maintaining and managing local genetic diversity for sustainable food and nutritional security [227]. 

### 2.5. Traditional Food Plants Provide Alternative Sources of Income to the Farmers and Unorganized Workers

In addition to having a key role in subsistence agriculture, as a source of food and medicine during shortages of food supply, they provide livelihood opportunities to rural communities [229,230]. Therefore, TFPs simultaneously act as a source of income for local communities. Among vegetables, *Cleome, Amaranthus, Corchorus* and *Vigna* spp. and fruit trees such as *Azanza garckeana, Adansonia digitata, Sclerocarya birrea, Strychnos spinosa, Vangueria infausta* and *Grewia* spp. are the major TFPs of Botswanan communities, providing them with income [231]. They grow naturally and the local women and children sell such crops or their products in formal and informal markets. This helps them raise their income—it may not be significant but can at least help them fulfill daily needs [231,232]. Cruz-Garcia and Price [233] reported that in the case of the poorest northeast region of Thailand, the sale of traditional food plants constitutes an important household income strategy to deal with situations of stress. Traditional crops such as *Eleusine coracana* (finger millet), *Vigna radiata* (green gram), *Coix lacryma-jobi* (Job’s tears), *Lens culinaris* (lentils), *Vigna radiata* (mungbean), *Sesamum indicum* (sesame), *Glycine max* (local soybean), *Ipomoea batatas* (sweet potato) and *Dioscorea* spp (yam) are the main source of income for poor and marginal farmers from East and South Asia [234]. In South Africa, traditional food plants are a vital source of income for indigenous communities [235], and in West Africa, the survival of small farmers in tribal communities is completely dependent on traditional food plants [236]. Secondary products of the TFPs are also highly marketable. For example, the malt produced from *Panicum sumatrense* (little millet) provides good incomes in India [237]. The processing of little millet led to generations of employment in the villages and increased the income of the rural folks significantly [196]. In India, it was reported that TFPs are a good source for increasing the incomes as well as improving the nutritional security of community people through the sale of several items such as ethnic millet papad, chakli, fermented breakfast food paddu and other novel foods prepared using little millet [238]. Islam et al. [239] reported that the poorest families in the Kurigram district of Bangladesh depend heavily on TFPs, especially in times of famine. Considering these limited studies, it can be stated that TFPs act as an alternative source of income for poor farmers and other poor communities including indigenous communities. 

## 3. Multi-Omics Tools to Dissect Nutritional and Stress-Related Traits for Ensuring Sustainable Global Food Security

Being traditionally and culturally important, TFPs are used across the globe for nutritional purposes by a large proportion of the population [59]. However, due to selective breeding, the yield and quality of TFPs is not up to the mark, and modern technologies can be used to improve yield and quality traits [240]. Advanced crop improvement tools can be implemented effectively to have a clear understanding of complex molecular machinery governing growth, development, nutrients, other quality traits and stress responses in TFPs [241]. The recent advancements and revolutions in omics technologies allow large-scale investigations of organisms at the gene, genome, metabolome, ionome and proteome levels at a faster rate within a relatively shorter period of time [242]. The chromosomal organization, sequence polymorphism and genome structure of the plants can be studied by using structural genomics tools and by developing genetic and physical maps of genomic regions controlling a particular trait of an organism [243]. Further, functional genomics technologies enable the understanding of the functions of genes regulating these traits [26,243]. Transcriptomics allows the study of the expression of total mRNA in a cell, tissue or in an organism under a given condition [244]. Transcriptomics also enable the identification of the transcripts and their correlation with the phenotypic data provides opportunities to decipher gene–trait relationships [244]. With the advancements in next-generation high-throughput sequencing technologies and the availability of advanced bioinformatics tools, it is easier to analyze large datasets including sequence alignment, annotation and expression profiling of genes [245]. Establishing a correlation of transcript abundance with the proteins and metabolites accumulation is slightly challenging because of the post-translational protein modifications and the regulation of metabolites by complex enzymatic pathways [246]. The quantitative and qualitative measurement of protein metabolite content is attained with the help of proteomics and metabolomics approaches [247]. Similarly, the complete mineral and elemental composition of a plant species can be understood with ionomics tools, and the integration of other omics tools such as genomics, proteomics and transcriptomics can help to establish the link between the elemental composition, transport and storage with the genes regulating various processes [248]. Omics tools are therefore very important for the discovery of the genes controlling a particular trait of interest in a crop plant [249,250].

**Figure 2:**
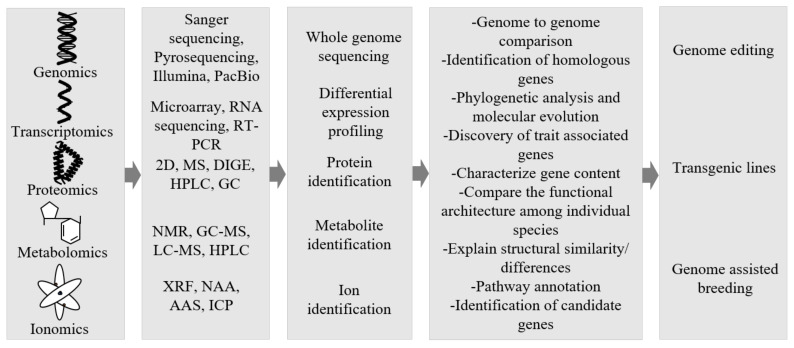
Multi-omics approaches to improve traditional food plants. Candidate genes governing important traits can be identified by combining the data from genomics, transcriptomics, proteomics, metabolomics and ionomics. Manipulations of candidate genes by various techniques to generate improved varieties [31,245,261].

In the past two decades, we have seen an increased number of plants being sequenced [251]. Arabidopsis was the first model plant to be sequenced and it has provided significant insights about the various processes in the plants. Completion of its sequencing took several years [252]. However, innovations and improvements in the sequencing technologies have made it possible to sequence large and complex genomes in a shorter period of time at lower costs [253]. Therefore, many of the genomes of major crops have been recently sequenced within a relatively shorter span of time [254]. Many studies have focused on the genome sequences of the model crop plants, but recently we have also seen the application of omics technologies to non-model crops [255,256]. To date, whole genomes of more than 328 vascular plant species (comprising 323 angiosperms, 5 gymnosperms and 3 lycophytes), 3 non-vascular land plant species (2 mosses and 2 liverworts) and 60 green algae have been sequenced [257]. Genome sequencing technologies provide a holistic overview of the various genetic components of an organism [258]. Whole-genome sequencing studies of plants have led to the identification of thousands of genes and other regulatory elements controlling the traits [259]. The integration of the low-cost sequencing technologies with computational bioinformatics tools and high throughput phenotyping technologies can enhance the identification of genes that govern important agronomic traits relevant to the production of food and its quality [260,261]. The results of multi-omics studies provide a holistic overview of the various genes, proteins, metabolomes and ionomes of the organisms. Therefore, the convergence of multi-omics technologies provides an important opportunity to accelerate the task of identification of genes that control agronomically relevant traits in plants, including traditional food plants, and speed up improvement programs using both conventional breeding as well as modern revolutionary CRISPR/Cas9-mediated and other gene editing technologies [28]. Figure 2 provides an overview of the application of omics and gene editing tools to the traditional food plants for their improvement.

The extension of integrative omics tools including whole genome sequencing to decipher the genetic and molecular basis of nutritional and stress-related traits in TFPs is not only crucial but also urgently required [31,37,255,262,263,264,265,266,267]. Table 2 presents some TFPs where omics tools have been applied successfully for the comprehensive dissection of important traits. The following subsections explain some important plants where omics tools have helped to understand the genomic basis of important traits in TFPs.

## 4. Examples of Application of Multi-Omics Tools to Traditional Food Plants

### 4.1. Lysine Biosynthesis in Amaranthus

One of the most important TFPs, also known as pseudocereal, is *Amaranthus* which belongs to the family Amaranthaceae. The genus *Amaranthus* comprises nearly 70 species [382,383]. *A. caudatus, A. cruentus* and *A. hypochondriacus* are three important species of *Amaranthus* that are traditionally consumed worldwide [384]. It is estimated that species of *Amaranthus* were domesticated nearly 8000 years ago in Central and South America and they sustained the Inca and the Aztec civilizations for several thousand years [385]. Unfortunately, the consumption of amaranth has reduced in modern times and only recently has there been an increased consumption of this species [386]. The growing interest in the consumption of amaranth has risen due to its unique nutritional composition [387]. *Amaranthus* is unique in its lysine content (5.19 g/16 g N) which has been found to be even more than that of milk [319]. This unique nutritional composition and resilience to a wide range of environmental conditions have led to its categorization as an important future, alternative wonder crop [388,389]. Amaranths are very important for another reason: they are C4 crops rather than most of the protein-yielding legume crops which are C3 plants [321]. Being C4, *Amaranthus* can perform better even at elevated temperatures when compared with the C3 species [321]. The nutritional and stress-resilient traits of amaranth have advantages which will definitely contribute to nutritional security. As the global temperature is rising, it is expected that such crops will provide more nutritional security to the growing population under elevated temperatures. Therefore, understanding the genetic basis of the nutritional and stress-resilient traits of *Amaranthus* is necessary. Lysine is important amino acid for human health, but unfortunately it is a limited in cereals, and this can be supplemented by consuming high-lysine-containing *A. hypochondriacus*. Sunil et al. [321] sequenced the genome and transcriptome of *A. hypochondriacus* and reported 24,829 protein-coding genes. This study further provided important details about the genes involved in the biosynthesis of betalains and lysine content [321]. The draft genomes of *A. tuberculatus*, *A. hybridus* and *A. palmeri* species were also reported recently by Montgomery et al. [390]. Taken together, these results will further enhance our genomic understanding of amaranths and trait manipulation.

### 4.2. Transcriptional Regulation of Anti-Nutritional Saponins in Chenopodium quinoa

Quinoa (*Chenopodium quinoa*) is an excellent nutritious grain that is designated as an important alternative future crop to improve global food security. Many genetic resources are not available for its improvement [391]. Jarvis et al. [392] reported the assembly of the reference genome sequence of quinoa. The genome sequencing has led to the identification of the transcription factor which may regulate the production of saponins, the anti-nutritional triterpenoids compounds synthesized in quinoa seeds. This is an important step towards establishing genetic resources for quinoa improvement [392]. Recently, Golicz et al. [393] performed genome-wide identification and analysis of orthologous genes of the *Arabidopsis thaliana* flowering genes in quinoa and provided important information about genes that controls vernalization, photoperiod, flowering and gibberellin biosynthesis pathway. The study further provided insights about the orphan genes that are unique to quinoa. This information is valuable as it will help to facilitate further programs aimed at quinoa improvement.

### 4.3. Genetic Mechanism of Stress Tolerance in Manihot esculenta

Cassava (*Manihot esculenta*) is a crop that is adapted to marginal soil conditions and erratic rainfall and is rich in carbohydrates and protein content [316]. Rabbi et al. [312] identified markers associated with the nutritional traits and have performed a genome-wide association mapping and identified candidate genes for carotenoid (*phytoene synthase*) and starch biosynthesis (*UDP-glucose pyrophosphorylase* and *sucrose synthase*) through this study. The transcriptomics study performed by Siirwat et al. [310] resulted in the identification of genes responsible for starch biosynthesis and revealed the mechanism behind the stress responses of cassava. Several transcriptomic studies on cassava have helped in unraveling the mechanisms of tolerance to various stresses. Utsumi et al. [314] reported upregulation of nearly 1300 genes during drought stress. The expression of Cu/Zn superoxide dismutase and catalase together during cold and drought stress improved drought and cold stress tolerance in cassava [394]. Lokko et al. [315] characterized heat stress transcription factors such as *A3* (*heat-shock transcription factor 21*) and *ATHB12* (a *homeobox-leucine zipper protein*) drought stress. In the same study, they reported expression of dehydration tolerance-related transcription factors such as *Early response to dehydration* (*ERD1*), *Responsive to dehydration 19* (*RD19*) and *Responsive to dehydration 22 precursor* (*RD22*) at the time of drought stress [315]. An et al. [317] reported drought-induced Di19-like protein during drought stress with the aid of proteomic tools. Wang et al. [395] reported the draft genome sequences of a cassava wild ancestor and a domesticated variety of cassava. This study led to the identification of gene models specific to the wild and domesticated varieties.

### 4.4. Genetic Dissection of Pathogen Resistance and the Early Fruit Development and Evolution in Physalis

The genus *Physalis* (groundcherry) belongs to the Solanaceae family. Several members of the Solanaceae family are important sources of food, spice and medicine. *Physalis ixocarpa*, *P. peruviana*, *P. pubescens* are underutilized berries that have many essential minerals such as potassium and vitamins such as Vitamin C [396]. They are also well known for the phenolic compounds which provide excellent antioxidant activity [397]. Much information on *Physalis* is not available and it is necessary to broaden the information about its nutritional content and other properties [340]. Garzón-Martínez et al. [398] studied the leaf transcriptome of *Physalis peruviana* and identified genes responsible for major biological processes and molecular functions. This study provided candidate genes responsible for resistance against diseases caused by viruses, fungi and bacteria. Even though tomato and ground cherry are in the same family, *Physalis* possess modified calyx, which is absent in *Solanum*. Gao et al. [399] studied the floral transcriptome of *Physalis* for the first time and identified some candidate genes causing variations accounting for the early fruit development and evolution in *Physalis floridana* and compared with *Solanum pimpinellifolium*. They reported a total of 14,536 single-copy orthologous gene pairs between them. It was revealed that the distinction between *Solanum* and *Physalis* was because of nine types of genetic variations that were differentially expressed either in trend or dosage at the flower–fruit transition between the two.

### 4.5. Detection of Genes Regulating Uptake and Storage of Micronutrients in Traditional Food Plants

Plants are an important source of a large number of mineral ions. Minerals and trace elements in optimal levels are very important for the growth and development of a plant and such minerals are a very important part of the human diet [400]. Plants acquire elements from the soil, fertilizers and manures. Therefore, soil type and composition influence the nutrient composition of the plants [401], and large-scale cultivation/adoption of TFPs in other regions may result in the change in their nutrient composition. However, studies by Akinola et al. [44] suggested that TFPs can increase the soil fertility (e.g., traditional legume species through nitrogen fixation). The cultivation of diverse traditional food plants also increases the soil organic matter than uniform crop cultivation [44]. TFPs can also be cultivated with low input on the marginal lands. Uptake of micronutrients from the soil and further transport within the plants is facilitated by several transporter proteins [402]. There are several metals that are toxic to plants as well as humans when consumed in higher concentrations. For example, excessive accumulation of aluminum, lead, zinc and cadmium results in metal toxicity which can harm the plants, and at times may result in the death of the plants, as well [403,404]. Their entry into the food web is also problematic as it may lead to serious health issues for humans. Therefore, quantitative determination of the total composition of such minerals and metals in edible plants is important for ensuring the safety of humans [405]. The total mineral and element composition of an organism has been termed as an “ionome” [248,406]. Ionome profiling of plants belonging to different species, collected from various habitats and cultivated in different soils can inform us about the fundamental differences in the total ionome composition [407]. Minerals such as sulfur, nitrogen and phosphorus are essential components of several metabolites, whereas trace metals such as zinc, copper, iron and manganese are essential components of several proteins [408]. Therefore, minerals and trace metals also regulate the composition of metabolites and proteins within the plants and perform important biological functions [408]. High-throughput techniques such as inductively coupled plasma mass spectroscopy (ICP-MS), inductively coupled optical emission spectrometry, X-ray fluorescence, neutron activation and atomic absorption spectroscopy analysis are nowadays employed to profile complete ionomes of plants [406]. Genomic technologies have enabled the identification of a large number of transporter genes and even gene families from model plants that facilitate mineral and metal uptake and transport in the plants [409]. A large number of indigenous communities still rely on TFPs, and the mineral and metal composition of TFPs greatly influences their health and well-being [410]. For example, an analysis of mineral and heavy metal contents of traditionally important aquatic plants of Tripura, India, was carried out by [411] using atomic absorption spectroscopy. Several other new studies have recently tried to investigate the nutritional composition of TFPs, which will have huge implications on future crop improvement and breeding strategies. For example, nutrient and antinutrient composition analyses of *Launaea cornuta, Vigna vexillata, Momordica foetida* and *Basella alba* performed by Chacha et al. [412] showed that they are rich in vitamin A, B1, B2, B3 and C and minerals such as Ca, Fe, Mg and Zn. The rich sources of micronutrients in the underutilized crops promise their capacity to abolish hidden hunger in the future. Combining results of ionomics with genomics can help in the detection of genes responsible for the accumulation of mineral elements in plants [413]. For example, Pasha et al. [282] uncovered the molecular mechanism behind the nutritional quality of Moringa plant parts. They reported genes responsible for mineral content including, vacuolar iron transporters (VIT), calreticulin for calcium storage, zinc transporters and magnesium transporters inside different tissues. Similarly, several calcium transporters such as *calcium ATPase*, *calcium exchanger* (*CaX*), *calcium-dependent protein kinase* (*CDPKs*) and *calcium-binding proteins* (*CBPs*) of *Eleusine coracana* (L.) were identified by Nirgude et al. [269] and Kumar et al. [271] with the aid of high throughput genomics tools. The identification of the plants with higher amounts of essential minerals and their genes would further enhance our understanding of the TFPs.

### 4.6. Unraveling the Mechanism behind High Amount of α-Linolenic Acid and Salinity Tolerance in Portulaca oleracea

Purslane (*Portulaca oleracea*) belongs to the Portulacaceae family. It is a highly nutritious vegetable with several medicinal properties [414]. It has been recognized as the richest source of a-linolenic acid, essential omega-3 and 6 fatty acids, ascorbic acid, glutathione, alpha-tocopherol and beta-carotene [415]. Because of exceptional quantities of omega-3 fatty acids in purslane, there is a growing interest to introduce this as an important vegetable crop [415]. Purslane is also considered as a future powerful biosaline food crop that can grow under various environmental stresses such as salinity, nutrient deficiency, heat and drought [416]. Liu et al. [339] quantified the fatty acid and β-carotene content of purslane with the aid of HPLC and GC. They reported 1.5–2.5 mg/g of fatty acid from leaves, as well as 0.6–0.9 mg/g and 80–170 mg/g from stems and seeds, respectively. Its leaves contain about 60% of α-linolenic acid (C18:3ω3) of total fatty acids. The β-carotene content in its leaves was recorded between 22 to 30 mg/g fresh mass. The first metabolite profile of *P. oleracea* was performed by Farag and Shakour [338] by using ultra-performance liquid chromatography-mass spectrometry on three taxa and recognized hundreds of metabolites including amino acids, phenolic compounds, alkaloids and fatty acids which indicate their nutritive and health benefits. Besides having an extraordinary nutrient profile, *Portulaca* shows excellent tolerance towards salinity stress and drought stress. The transcriptome sequencing and metabolome analysis of *P. oleracea* regarding salinity tolerance were conducted by Xing et al. [417]. They reported that genes of photosynthesis and aquaporins were depressed during salinity treatment which indicates the inhibition of photosynthesis and water uptake during salinity stress. However, the expression of L-3-cyanoalanine synthase/cysteine synthase and cyanoalanine synthase were elevated. Higher content of pyrophosphate, D-galacturonic acid and elaidic acid was detected in salinity-tolerant plants that positively regulate glycolysis, energy supply and integrity of cell membrane. These studies regarding nutritional profiling and genes that regulate the tolerance to salinity are important for further improvement programs.

### 4.7. Higher Accumulation of Lycopene in Elaeagnus

Silverberry (*Elaeagnus*) belongs to the Elaeagnaceae family which is recognized as an important fruit crop used widely because of the presence of high lycopene content in the berries, which is ten times higher than tomatoes, especially in the species *E. umbellata*. [65]. The berries are well known for their high ascorbic acid, protein and magnesium content, as well as drought tolerance and adaptation to a variety of moisture and edaphic conditions [418]. The proteomic study of *E. umbellata* with special emphasis on fruit quality traits was performed and the quantity of soluble sugar, organic acids, lycopene and total protein content was analyzed [361]. The expression of the *phytoene synthase* (*EutPSY*) gene was found to be correlated with the higher accumulation of lycopene in *E. umbellata,* suggesting its importance [360]. The results suggest that the *EutPSY* gene could be considered as a target for increasing the lycopene content in other fruits and hence increase their quality.

### 4.8. Nutritional Composition of Dioscorea, a Neglected Staple Tuber Crop of the Indigenous Communities

Yam (*Dioscorea*) is one of the oldest tuber crops harvested from the wild in the tropical regions throughout the world and acts as a chief food item for a number of indigenous groups [332]. Yam is the main source of diosgenin-steroid which is effective against neurodegenerative diseases [419]. It is also an effective nutritional supplement with a high amount of protein. There are about 600 *Dioscorea* species, but only seven contribute to the human diet in the tropics [420]. Despite its wide utility, this tuber crop remains orphaned and its genomic and proteomic information is not available in detail [421]. Recently, little progress on genomic studies of *Dioscorea* have been reported. Nakayasu et al. [422] performed comparative transcriptome analysis of high-saponin-containing yams, i.e., *D. esculenta* and *D. cayenensis*, and low-saponin-containing *D. alata* for understanding biosynthesis of steroidal saponins and identified the *β-glucosidase* (*DeF26G1*) gene to be responsible for higher accumulation of saponins in *D. esculenta.* The first report of genome-wide characterization of *Dioscorea* taxon was reported in *D. zingiberensis* by Zhou et al. [423] where they identified 4935 genes, 81 tRNAs, 661 miRNAs and 69 rRNAs. Transcriptome profiling of *D. alata* led to the identification of several thousand unigenes, some of them code for enzymes involved in the flavonoid biosynthesis pathway. The study further found the upregulation of several genes such as *flavanone 3-hydroxylase* (*F3H*), *chalcone isomerase* (*CHS*), *dihydroflavonol 4-reductase* (*DFR*), *leucoanthocyanidin dioxygenase* (*LDOX*), *flavonoid 3′-monooxygenase* (*F3′H*) and *flavonol 3-O-glucosyltransferase* (*UF3GT*) in the tubers of purple flesh cultivar compared to white flesh cultivar [397]. Price et al. [332] performed whole metabolite profiling of yam and identified 152 metabolites. They developed biochemical phenotyping of accessions of the yam varieties through a large-scale metabolomic study. The integration with other omics studies can be used for yam breeding programs.

### 4.9. Transcriptional Basis of Lipid Biosynthesis in Salvia, a Wonder Seed for the 21st Century

Some species of the genus *Salvia* such as *S. columbariae, S. hispanica* and *S. polystachya* are commonly known as chia and are consumed for their seeds which have multiple nutritional and medicinal benefits [424,425]. Chia seeds are rich in insoluble fiber, high omega-3 and omega-6 fatty acids, *α*-linolenic acid, linoleic acid, proteins, amino acids, antioxidants and minerals [426,427]. Because of their high nutritive value, chia is known as the “seed for the first 21st century” [426]. The seeds of chia also contain metabolites that show anti-cancer, anti-inflammatory, antioxidant, anti-blood clotting and antidiabetic activities. The seeds have also been found to show action against cardiovascular diseases and hypertension [427,428,429,430]. The transcriptomic study of wild and cultivated accessions of *S. hispanica* suggests the genetic basis of oil and protein content accumulation in chia seeds [431]. The study has also identified several transcription factors such as *AP2/EREBP202* and simple sequence repeat (SSRs) markers which would be helpful for breeding or in translational genomics programs. The transcription factor *AP2/EREBP* is known to regulate the expression of genes related to fatty acid biosynthesis [431]. Transcriptome analysis of chia seeds from its different developmental stages has further identified important candidate genes such as *monoacylglycerol acyltransferase* (*MGAT*), *Acyl-CoA desaturase 1* (*OLE1*), *diacylglycerol acyltransferase* (*DGAT1, 2* and *3*), *phospholipid:diacylglycerol acyltransferase* (*PDAT*), *Thiolase* and *Desaturase,* responsible for lipid biosynthesis and oil accumulation [432].

### 4.10. The Adansonia digitata Contains More Vitamin C Than Oranges

The *Adansonia digitata* L. is commonly known as African baobab and belongs to the family Malvaceae. It is a very important tree with multiple benefits and is a source of traditional food in Saharan countries [433]. Additionally, it is also a source of medicine, fiber and income for rural communities [434,435]. Almost all its parts can be consumed and it contains high vitamin C content as compared to oranges [434]. Using microsatellite loci, Chládová et al. [435] suggested huge genetic diversity among its populations. However, further research is needed to understand the genetic basis of the higher accumulation of vitamin C and other important compounds that make it a wonder tree.

## 5. Integrating Omics and Gene Editing Tools for Improvement/Domestication of Traditional Food Plants

A lot of information is available on the genetic regulation of yield, nutritional quality and stress-related traits of several model domesticated crops [436,437,438]. The genetic and genomic analysis of many domesticated crops such as maize, tomato, rice, sorghum and wheat have led to the identification of several genes/QTLs that regulate domestication traits [436,437,439,440]. Some of the important domesticated crops, their relative traditional crops and the genes regulating domestication traits are shown in Table 3. The results of genomics and other omics research have provided fundamental clues about the genetic regulation of important traits [441]. The knowledge obtained using omics approaches can be used for crop improvement programs such as the development of nutritionally superior, disease-resistant and stress-tolerant crops with high yields [241]. The integration of genomics with gene editing tools is now possible, and allows editing of important genes with greater precision, accuracy and rapid pace [442]. Finding plants with desirable traits and having superior traits is an important step [443]. The plants with one or more of the desirable traits such as superior nutritional composition, high yields and biotic and abiotic stress tolerance should be given priority [444]. With the development of both genomics tools and bioinformatics pipelines, it is now easier to identify the genetic variation in wild species, which can be utilized for the transfer of traits to accelerate adaptive introgression in crops, as well as de novo domestication of wild relatives and landraces [83]. Since much genomic information is available on domesticated crops and other model plants, it is now possible to directly translate this information to the non-model TFPs for their rapid improvement by using various gene editing tools such as mega nucleases, Zinc Finger Nucleases (ZFNs), Transcriptional activator-like Effector Nucleases (TALENs) and Clustered Regularly Interspaced Short Palindromic Repeat-Associated Protein 9 (CRISPR-Cas9) [445,446,447,448]. Among the several gene editing tools, CRISPR-Cas9 has been one of the most important and popular gene editing tools and has attracted considerable attention from crop scientists [20,215,443,447,449,450]. The CRISPR-Cas9 editing has increased possibilities for genome modification and enables metabolic engineering, biofortification and crop improvement [443,444,449]. Several attempts for improving various traits such as yield and stress tolerance in several crops have been exercised using CRISPR/Cas [443].

**Figure 3:**
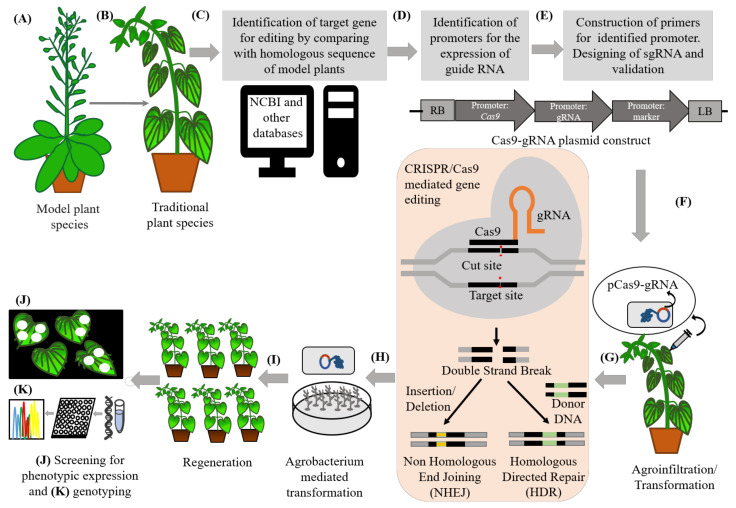
General workflow of CRISPR/Cas9 based gene editing in neglected crops for their improvement. (**A**) Extensively studied model plant species chosen for the ease of identification of homologous genes governing particular traits. (**B**) Underutilized, orphan or neglected traditional plants with undesirable traits can be edited for trait improvement, and biotic and abiotic stress tolerance. (**C**) Identification of target gene(s) for construction of sgRNA by comparing with a homologous sequence of model plants using databases such as the National Center for Biotechnology Information. (**D**) Identification of promoters for the expression of guide RNA. (**E**) Construction of plasmid carrying Cas9, gRNA and reporter gene in the promoter region with software Cas-Designer. (**F**) Agroinfiltration on young leaves with Agrobacterium harboring the construct Cas9-gRNA-Reporter. (**G**) Induction of precise breaks at the target sequence site(s). Endogenous machinery of cells repairs the breaks by non-homologous end joining (NHEJ) in the absence of a homologous repair template resulting insertions/deletions (indels) that disrupt/change/edit the target sequence or homology directed recombination by providing a homologous repair template, thereby inducing genomic mutations at the target locations. Other than CRISPR/Cas9 zinc finger nucleases (ZNF), mega nucleases and transcription activator-like effector nucleases (TALEN) are also used for gene editing, but the feasibility of CRISPR/Cas9 is greater when compared with other methods. (**H**) Validation of the efficiency of CRISPR/Cas9 for targeted mutagenesis in stable transgenic plants. The construct pCas9-gRNA-reporter introduced into nodal-explants after tissue culture using the Agrobacterium-mediated transformation method. (**I**) Regeneration of stable transgenic plants. (**J**) Screening of the regenerated plants for the mutated effect by checking their phenotypes. (**K**) Genotyping or putative transgenic plants containing Cas9 confirmed by PCR analysis [447,455,458].

The CRISPR-Cas9 mediated gene editing is based on the guidance of short RNA sequences termed as guide RNAs which are designed to complement target DNA [451]. The target DNA is cleaved by a Cas endonuclease that results in a single or double-strand breaks in the DNA [451], followed by ligation of the DNA by the endogenous repair mechanisms [452,453,454]. In case of gene editing of less-studied plants, for the identification of particular traits and related genes, homologous genes from extensively studied plants such as model plants are used. The genetic information from the domesticated species can be translated to the traditional food plants. (See next section for the example of translation of genetic information from *Solanum lycopersicum* and *S. pimpinellifolium* to *Physalis pruinosa.*) With the help of databases such as the National Center for Biotechnology Information (NCBI), identification of target genes for their construction of sgRNA by comparison with a homologous sequence is possible [455]. Software are used for the construction of plasmid that carries Cas9, gRNA and reporter genes along with their promoter [456]. Cas-Designer is good software for this purpose [456]. For delivering the construct Cas9-gRNA-Reporter, several methods such as agroinfiltration and electroporation can be used [457]. After delivery, induction of precise breaks at target sequences takes place at the target site. Endogenous machinery of cells repairs the breaks by non-homologous end joining (NHEJ) in the absence of a homologous repair template that results in insertions/deletions (indels) that disrupt/change/edit the target sequence or homology directed recombination (HDR) by providing a homologous repair template thereby inducing genomic mutations at the target locations [447,458]. For the validation of CRISPR/Cas9, editing the construct pCas9-gRNA-reporter is introduced into nodal explants after tissue culture using the *Agrobacterium*-mediated transformation method. After the regeneration of successful transformed plants, phenotypic and genotyping (using RT-PCR) and screening help to check the mutation effect [455]. A generalized workflow involving various steps in genome editing for improved varieties is presented in Figure 3.

## 6. Recent Successful Examples of Gene Editing and Translational Genomics in Traditional Food Plants

**Figure 4:**
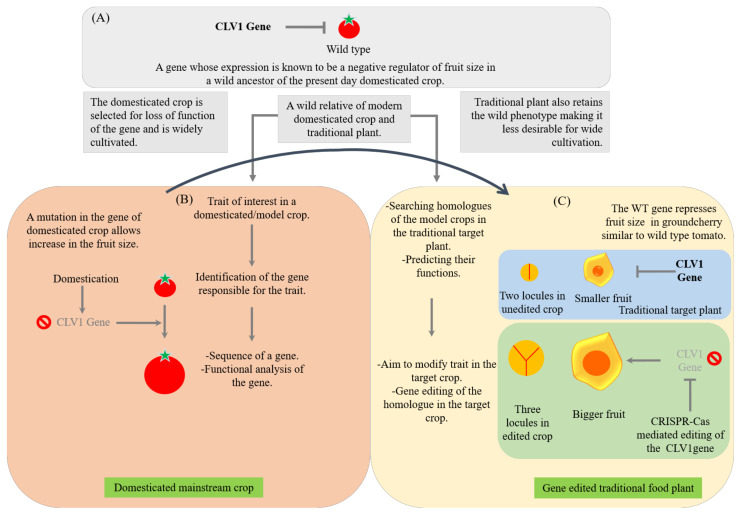
Example of rapid improvement of a traditional orphan food crop for larger fruits. The genomics information obtained from the tomato (a domesticated crop) genome sequencing and the functional analysis of the genes is directly translated to the traditional crop, groundcherry (a traditional food plant) [446]. A wild type gene *CLV1* which is a negative regulator of fruit size in the domesticated tomato (**A**), a mutation during domestication has occurred in this gene leading to the formation of a bigger fruit size of domesticated tomato (**B**) and its homologue in a traditional food plant, groundcherry is targeted for gene editing for its improvement for bigger fruits (**C**).

TFPs with many beneficial traits are important for a sustainable food system. *Physalis pruinosa* (groundcherry) is a traditionally important plant consumed in various parts of the world for its important nutritional properties [340,511]. Huge inter- and intraspecific diversity of *Physalis* is available in the world, but it is not cultivated or consumed on a larger scale because of its certain undesirable traits such as extensive growth habit, smaller fruits and fruit dropping because of an abscission [446]. It is a relative of the tomato as both of them belong to the family Solanaceae and they share common genetic architecture with the same chromosome number of 12. Since both are from the family Solanaceae, and we know a lot about the genetic regulation of various traits in tomato, it is easy to translate genetic information from the model tomato to the non-model traditionally important crop, groundcherry for its improvement using gene editing tools [441,446]. Gene editing tools can be used to rid of undesirable traits from ground cherries. On these lines, a study was carried out by Lemmon et al. [446] and they obtained very successful gene-edited crops with improved characters in groundcherry. The undesirable characteristics of *Physalis* are similar to the wild ancestor of the tomato, *S. pimpinellifolium,* which underwent domestication in its traits leading to modern-day *S. lycopersicum*. Using gene editing, Lemmon et al. [446] targeted repressors of the florigen pathway to increase flower numbers and delimit flowering time, both on primary and axillary shoots. They performed a knockout of classical *SELF PRUNING* (*SP*) genes that control determinate and indeterminate growth habits of the plant. The results led to extreme compactness in *P. pruinosa.* Another knockout of the florigen repressor, *SELF PRUNING 5G* (SP5G), resulted in increased axillary flowering and fruit density. Targeting of the shoot apical meristem size-regulating gene *CLAVATA* resulted in increased flower meristem size, additional flower organs and conversion of small two-loculer fruit into larger three-loculer fruit, as illustrated in Figure 4 [446]. This study has opened up new hopes and possibilities for the rapid improvement and fast domestication of traditional orphan and wild crops. Many other groups around the globe are now focusing on editing the genes in non-model crops based on genetic and genomic information obtained from model crops [68,441]. The gene editing tools are particularly employed with an aim to increase quality, enhance yields, improve biotic and abiotic stress resistance and expand geographical ranges of cultivation of traditional orphan crops [446]. However, TFPs have not undergone intensive selection for domestication [512]. Thus, traditional orphan crops are less productive and unsuitable for cultivation at larger agricultural scales [52]. Similar studies can be undertaken and the information from omics studies can be combined with gene editing tools to other TFPs. A similar approach can be extended to wild edible species for de novo domestication [68]. The de novo domestication of wild plants is considered as an important solution for designing customized crops for the future [68]. By unleashing the multiplexing ability of CRISPR/Cas9 technology, multiple targets can be modified simultaneously in an efficient way by pyramiding multiple beneficial traits [452]. Taken together, the results of these studies suggest that the gene editing tools are a valuable tool to target homologs of domestication genes in traditional food plants quickly [20].

Gene editing has led to several revolutions in the field of crop improvement and it has been realized in several major crops and other plants such as tomato, maize, tobacco, grapevine, apple, opium poppy, cucumber and cotton for important traits and the results obtained are impressive [513,514,515,516,517]. Zsögön et al. [445] engineered *S. pimpinellifolium* (wild) using CRISPR/Cas9 and their several traits were altered that resulted in superior gene-edited *S. pimpinellifolium* than the *S. lycopersicum*. In 2014, CRISPR/Cas9 gene editing was successfully applied to tomato and citrus. Some successful cases of CRISPR/Cas9 fruit trait improvement are cucumber, apple, grape (2016), watermelon (2017), kiwifruit, banana, cacao, strawberry, papaya and groundcherry [449]. Other examples of successful gene editing using CRISPR/Cas9 include trait improvement of grain number, grain size, panicle architecture of rice [518,519], grain length, weight of wheat [520], seed oil composition (high oleic and low polyunsaturated fatty acids) of flax [521], late-flowering in soybean [522], reduced zein protein in maize [523]. Most of the successful works, however, are reported in major crops, and efforts are needed to improve and mainstream TFPs with the aid of genome editing tools and integrative genomics approaches. Examples of successful gene editing in crops to date are included in Table 4. Varshney et al. [524] explained the success story of translational genomics of the grain legume crops chickpea (*Cicer arietinum*), common bean (*Phaseolus vulgaris*), groundnut (*Arachis hypogaea*), pigeon pea (*Cajanus cajan*) and soybean (*Glycine max*) for their drought tolerance and pathogen resistance by multiple QTLs or genes from model legume *Medicago truncatula*. Ji et al. [525] attempted gene editing using CRISPR/Cas9 in Cowpea (*Vigna unguiculata*) which is also an important traditional food plant because of its symbiotic nitrogen fixation capability. Recently Syombua et al. [455] introduced a CRISPR/Cas9-based genome editing system for underutilized yam *Dioscorea alata* with improved genetic transformation, which can lead to trait improvement in yam. By the establishment of an efficient CRISPR/Cas9 editing protocol, Syombua et al. [455] suggested that it is possible to remove undesirable traits of *Dioscorea alata* such as poor seed set and non-synchronous flowering. Considering the importance of gene editing technology and its application in successfully editing genes of several crops for improved varieties and the beginning of editing traditional orphan crops, future studies aiming at the extension of this technology will lead to the mainstreaming of many TFPs. It will lead to diversification of the food basket of people across the globe, reducing excessive reliance on a select number of crops.

## 7. Challenges to Translational Genomics Using Gene Editing Technology/Tools

Although considerable progress has been achieved in the field of translational genomics particularly with the aid of gene editing tool CRISPR/Cas9 [552], there are also a number of important challenges. Several traits are quantitatively controlled and require multiple genes. Therefore, to produce desired phenotypes in the edited crops, we need to edit multiple genes [450]. Further, genomic information of many traditional food plants is not available. Another important challenge is that it is not easy to create precise modifications in DNA sequences. However, several gene editing strategies such as replicons, base editors and targeted nonhomologous insertions provide efficient precise gene editing in plants [457]. The unavailability of efficient delivery methods for gene editing reagents (DNA plasmid, mRNA (Cas9 + sgRNA), Ribonucleoprotein (RNP)) is another challenge [457]. Several other challenges such as ethical issues and technical bottlenecks are discussed elsewhere (see [450,552,553,554]).

## 8. Conclusions and Future Perspectives

Many TFPs have been a part of human civilizations since ancient times. Different parts of the plants are consumed by humans from generation to generation in different geographical areas of the world. They are unique as they possess various nutritional components and abiotic stress tolerance-related traits. Several studies have shown that some TFPs such as quinoa, millet, cassava and amaranth show tolerance to multiple abiotic stresses. The nutritional composition of many TFPs is also incredible, with a variety of health benefits and pharmacological values. Multi-omics tools have been applied to several TFPs for unraveling the basis of important traits. The availability of genome sequence information of relatives can be directly translated to many TFPs using several tools including CRISPR/Cas-mediated gene editing. Many TFPs are grown regionally and have regional importance. Therefore, they have undergone some level of domestication, and if they have to be domesticated and cultivated at a large scale, it is essential to get rid of undesirable traits that burden these crops. Since they are subjected to a certain level of domestication, tweaking a few genes using gene editing technologies will make them cultivable at a large scale, as evidenced by studies on groundcherry by Lemmon et al. [446]. The reintroduction of improved traditional crops into the current food systems will help diversify the food basket of the public, giving more options. Identification and mainstreaming of traditional food plants having higher nutritional and micro-nutritional values will help eradicate hidden hunger, which is prevalent due to the deficiency of the micronutrients in diets [51,555]. One of the issues linked to mainstreaming TFPs is their increased demand in food-secure regions due to scientific studies and increased popularity suggesting their health benefits, as well as increases in their prices. The increased prices could increase the income of the local farmers and the communities that rely on them. The increased demand for traditional foods also means increased opportunities in the entire supply chain from production, distribution and marketing to delivery for consumption. However, the increased prices may be beyond the purchasing capacity of the poor farmers and consumers in the producing regions. This increased popularity and increased demand has led to skyrocketing prices of quinoa in the Andes, Bolivia, and as a result, local farmers have resorted to non-traditional foods [556]. This situation has led to a situation where growers are in a dilemma of whether to have traditional foods or non-traditional foods. Another study by Enrico Avitabile in collaboration with the FAO suggests that increased prices of quinoa led to increased economic power of the local farmers in Bolivia [557]. He argues that although it led to overall reduction in the domestic consumption among the rural population, the increased incomes increased their economic freedom to access richer diets. Similar situations may also arise with similar TFPs if they become more popular and their increased demand elsewhere affects food and nutritional security in the regions where such traditional crops are produced. That will be an unhealthy situation and steps must be taken to ensure that the real producers of TFPs also consume healthy traditional foods for their own nutritional security, and not just remain producers.

## Figures and Tables

**Table 1:** Diverse traditional food plants grown across the globe, their uses and nutritional importance.

Sl. No.	Traditional Food Plant	Occurrence and Traditional Use	Important Nutritional and Stress Resilient Traits
1	*Lolium perenne* (Perennial ryegrass, Poaceae)	Used as a cereal in North America, Southern countries of Europe, North Africa, Middle East and towards the eastern sides of Central Asia [84].	The seed has a nutritional value similar to oats (*Avena sativa*) and contains gluten which is an important trait of baked food [84].
2	*Cleome gynandra* (Stinkweed, Capparaceae)	It is an important vegetable in rural areas of several African countries [85].	Rich in linoleic acid and amino acids content such as glutamic acid, aspartic acid, arginine, tyrosine, histidine and lysine [85]. The C4 photosynthetic pathway helps them to survive in dry and hot conditions [86]. Adapted to several types of soils and can grow in humid, semiarid and arid climates [87].
3	*Basella alba* (Vine spinach, Basellaceae)	Used throughout temperate regions and the tropics [88].	Leaves are rich in calcium, fiber, fat, protein and carbohydrates [89]. They are extremely heat tolerant and are also adapted to a variety of soils and climates [90].
4	*Vigna subterranea* (Bambara groundnut, Fabaceae)	An important indigenous crop in sub-Saharan African countries such as South Africa, Senegal and Kenya, and Madagascar [91].	Drought and pest resistant, able to survive in poor soils. Rich in protein whereas fat content is low [92]. Rich in essential sulfur-containing amino acids such as Methionine and provides a good amount of fiber, iron, potassium and calcium [93].
5	*Chlorophytum comosum* (Spider plant, Asparagaceae)	Iran [94].	Tubers are rich in carbohydrates, fiber and calcium [94].
6	*Corchorus* spp. (Mallow, Malvaceae)	In India, Africa and the Middle East, it has been a popular vegetable since ancient times [95].	The leaves are a good source of calcium, iron, beta carotene, vitamin C and α-tocopherol. Plants also show antioxidant activity [96].
7	*Macrotyloma uniflorum* (Horse gram, Fabaceae)	Cultivated in Asian countries, especially India and Myanmar, and African countries [97].	Adapted to drought and poor fertile solid conditions. A potential source of nutrients such as protein, iron and calcium [97].
8	*Fagopyrum tataricum*, *F. esculentum* (Buckwheat, Polygonaceae)	Found on a large scale in Asian and Southeast Asian countries. It was spread from China to Japan and Korea. It is also consumed in Russia, Sweden, Europe and North America [98].	Proteins are rich in essential amino acid lysine [98].
9	*Brassica carinata* (Ethiopian mustard, Brassicaceae)	Consumed all over the world and considered important food crops in European countries, India, Japan and China [99]. It is an important green leafy vegetable in Zambia and in most parts of tropical Africa [100].	High levels of glutamic acid, arginine and proline [99].
10	*Colocasia esculenta* (Taro, Araceae)	It is found all over the Pacific islands and other parts of the world. Africa is the bulk producer of taro, followed by Asia and Oceania [101].	Rich in small starch grains and proteins. Nutritive than other tubers and rich in vitamins (thiamine, vitamin C, niacin and riboflavin) and minerals (iron, phosphorus and calcium). Taro corms have a high quantity of magnesium and potassium; also a good source of carotene [102].
11	*Boscia senegalensis* (Aizen plant, Capparaceae)	Native to the Sahel region of Africa [103].	Protein contains a considerable quantity of tryptophan and arginine. Zinc and iron are present at a relatively high level [104]. High degree of drought resistance [105]. It is highly drought tolerant and can perform very well poor soil conditions [103].
12	*Sphenostylis stenocarpa* (African yam bean, Fabaceae)	Cultivated in different regions of African countries [106].	The legume and tuber of the plant is edible. Adapted to wide range of climatic, geographical and edaphic conditions [106]. They have a short growing period [107].
13	*Telfairia occidentalis* (Fluted guard, Cucurbitaceae)	The crop is extensively cultivated in southern Nigeria [108].	Leafy vegetable with oil-rich leaves. Its nutritious seeds are also consumed as they are a good source of minerals and proteins [108].
14	*Digitaria exilis* (Fonio millet, Poaceae)	Cultivated throughout West Africa [109].	Rich in minerals, vitamins, carbohydrates, protein, fiber and iron. Another advantage is that it is gluten free [110]. Grows in poor-fertile soil and rain-deficient areas [111]. Long storage life without preservatives [109].
15	*Crotalaria brevidens* (Rattle pod, Fabaceae)	Widely consumed and cultivated in East Africa and West Africa [112].	Good source of β-carotene, ascorbate, folic acid, riboflavin, iron, calcium and magnesium [59]. They have nitrogen fixing capacity, drought tolerance, produce seeds under tropical conditions and are suitable for intercropping [112].
16	*Dacryodes edulis* (African pear, Burseraceae)	Cultivated in Guinea and widely in other tropical parts of Africa [113].	Edible fruits contain lipid, protein, vitamins and minerals such as potassium, calcium, magnesium, iron, zinc, copper and selenium [113,114,115].
17	*Treculia africana* (African breadfruit, Moraceae)	Cultivated in Nigeria and Africa as a whole [116].	Seeds are highly nutritious because of the presence of minerals such as potassium, magnesium and calcium, vitamins, fats, proteins and carbohydrates [117]. Grows in marginal areas where other species may not be able to grow [116].
18	*Momordica balsamina* (Balsam apple, Cucurbitaceae)	Indegenous to the countries of tropical Africa, Arabia, Asia and Australia. Widely distributed in Swaziland, Namibia, Botswana and the provinces of South Africa [118].	Leaves are rich in protein and fat. They have higher values of minerals such as calcium, magnesium and iron [119]. Leaves also contain 17 amino acids [118].
19	*Adansonia digitata* (Baobab, Malvaceae)	Distributed throughout the drier parts of Africa, Namibia, Ethiopia, Sudan and Sahara [120].	Contains vitamin B2/Riboflavin, calcium, phosphorus, iron, vitamin A and vitamin C. It contains almost 10 times more vitamin C than oranges [121]. It is drought tolerant and can tolerate various ranges of pH. It can also grow in calcareous soils and rocky hillsides [120].
20	*Berchemia discolour* (Bird plum, Rhamnaceae)	Indigenous Southern African fruit tree species. Widely distributed in the regions of northern, eastern, central and southern Africa [122].	The dry pulp is a rich source of calcium, carbohydrates, iron, sodium, potassium and magnesium [122].
21	*Heinsia crinita* (Bush apple, Rubiaceae)	Indigenous to West Africa, especially the southern part of Nigeria [123].	Rich in calcium, magnesium, potassium, iron and zinc [123].
22	*Psophocarpus tetragonolobus* (Winged beans, Fabaceae)	It grows widely in Malaysia, Indonesia, the Philippines, Bangladesh, Thailand, Sri Lanka, India, Myanmar and African countries [124].	Seeds, pods, tubers, foliage and flowers are nutritious [124] and contain higher crude protein [125]. It has adequate quantity of minerals such as P, K, Ca, S, Na, Mg, Mn, Fe, B, Sr, Zn, Ba, Cu and Cr, and vitamins such as vitamin A, vitamin B1, vitamin B2, vitamin B3, vitamin B6, vitamin B9, vitamin C and vitamin E [126]. It is suitable to be grown in hot, humid conditions and possess nitrogen fixation capacity [127].
23	*Tropaeolum tuberosum* (Mashua, Tropaeolaceae)	Traditional subsistence tuber crops indigenous to the Andean highlands [128].	It can be grown in poor soils without pesticides and fertilizers [128]. They have a high level of protein with an ideal balance of essential amino acids. More content of vitamin C and provitamin A (equivalents of Retinol) than other Andean tubers. Rich in magnesium, phosphorus, iron and zinc [129].
24	*Oxalis tuberosa* (Oca, Oxalidaceae)	Second important tuber crop in Bolivia and Peru. Cultivated as an important crop in Central Andes, Chile, Argentina, Ecuador, Bolivia and Peru [130].	Iron- and calcium-rich tubers [131]. Notable quantities of fructo-oligosaccharides reported [130].
25	*Smallanthus sonchifolius* (Yacon, Asteraceae)	Cultivated in Bolívia, Peru, Czech Republic, Argentina, Italy, Brazil, Ecuador, Korea, Japan, New Zealand and the United States [132].	Rich in fructooligosaccharides that are good for colon health. They are extremely hardy plants and adapted to cold and hot conditions [133].
26	*Chenopodium pallidicaule* (Cañiwa, Amaranthaceae)	Majorly grown in Bolivian and Peruvian Altiplano [134].	Exceptional protein quantity and quality and grains are enriched with micronutrients such as calcium and iron [134]. The nutritional value is equivalent to milk proteins [135]. Gross et al. [136] recognized that it has a balanced amino acid composition and 15.3% protein content. It does not have saponins, which gives a bitter taste and hence it is possible to consume directly without washing. Drought- and frost-resistant plants, well adapted to rocky and poor nutrient soil [134].
27	*Lablab purpureus* (Hyacinth bean, Fabaceae)	Third high priority vegetable in the south-western and central regions of Bangladesh [137]. Cultivated as a minor crop in tropical regions of Asia and Africa [138]	Extremely resilient to drought-prone areas. A good source of vegetable protein and also a potent source of fats, carbohydrates, fibers and minerals such as phosphorus, calcium and iron [139].
28	*Sclerocarya birrea* (Marula, Anacardiaceae)	African fruit tree [140].	Seeds contain sufficient amounts of calcium, phosphorus, magnesium, iron, potassium and copper. Seed edible part has 36.4% of protein, with high levels of cysteine and methionine. Fruits are rich in ascorbic acid and juice extracts contain 33 types of sesquiterpene hydrocarbons [140].
29	*Amorphophallus paeoniifolius* (Elephant foot yam, Araceae)	Cultivated in Southeast Asian countries such as Malaysia, the Philippines and Indonesia [141].	Multiple edible parts such as leaves, rhizomes and petioles. Immunity booster and rich in carbohydrates, phenols, alkaloids, tannins, flavones, steroids, coumarins, vitamins, minerals and antioxidants [142].
30	*Solanum quitoense* (Lulo, Solanaceae)	Majorly cultivated and consumed in Columbia, Ecuador and Central America [143].	Carotenoid content of fruit is high. Very low fat content but rich in proteins [143].
31	*Senna tora* (Sickle pod, Caesalpiniaceae)	India [144].	Its leaves consist of lipids, crude fiber, crude protein and minerals (iron, calcium, cobalt sodium, zinc, magnesium, manganese and potassium) [144]. Sickle pods hold great potential as a source of medicine, minerals. They exhibit drought tolerance [145].
32	*Ziziphus jujuba* (Buckthorns, Rhamnaceae)	Widely distributed in Europe, Southern and Eastern Asia and Australia [146].	They grow in different soils and are resistant to alkalinity and salinity, and better adapted to arid regions. They contain high amounts of fructose and fiber. Jujube fruit is rich in unsaturated fatty acids especially linoleic acid (omega-6). They are rich in vitamin C also. Excellent source of magnesium, phosphorus, potassium, sodium and zinc [146,147].
33	*Pyrus pyrifolia* (Asian pear, Rosaceae)	It is cultivated throughout Central and South China, Russia, Korea, Japan, Vietnam, Thailand, India, Indonesia and the Philippines. As of recently, it is also cultivated in Australia, New Zealand, the USA and Europe (Italy, France) [148].	Abundant in vitamin B and minerals [148].
34	*Achyranthes bidentata* (Ox knee, Amaranthaceae)	Grown as cereal in Korea, Vietnam and China. In India and China, leaves and seeds are consumed [149].	Seeds are rich in proteins and minerals such as iron, calcium, phosphorus, potassium and magnesium. It contains 1.6 times higher quantity of vitamin E than Amaranthus seeds [149].
35	*Setaria italica* (Foxtail millet, Poaceae)	China, India and other Asian countries [150].	Great tolerance to drought and can grow in arid and barren lands [150].
36	*Grewia asiatica* (Phalsa, Malvaceae)	Various parts of South Asia including Cambodia, Philippines and Laos [151].	Rich in vitamin A, vitamin C, minerals and fiber. Can grow nicely under water-deficient conditions [152].
37	*Aegle marmelos* (Bael, Rutaceae)	Cultivated throughout India, Nepal, Tibet, Sri Lanka, Laos, Thailand, Malaysia, Phillipines, Vietnam and Myanmar [153].	Potent source of vitamins (A, B, C, folate) and minerals, antioxidants, dietary fiber, amino acids and bioactive compounds [153]. They are adapted to high salinity conditions [154].
38	*Carissa carandas* (Koranda, Apocynaceae)	India [155].	Rich source of vitamin C, iron, calcium and phosphorus [155]. They are xerophytic and suitable for growing in dry land [156].
39	*Artocarpus heterophyllus* (Jackfruit, Moraceae)	Majorly cultivated in tropical regions of Burma, Sri Lanka, Indonesia, Malaysia, Jamaica, India, Mauritius, Brazil, East Africa, Seychelles and Rodrigues Island [157].	Fruits are rich in carbohydrates and vitamins such as A, C and folic acid. Rich in calcium and magnesium [158]. Tolerant to water deficit conditions [157].
40	*Ullucus tuberosus* (Olluco, Basellaceae)	Peru, Ecuador, Colombia, Venezuela and northwestern Argentina [159].	Resistant against frost and drought and can perform in poor soils. Lower in fat than corn [159].
41	*Arracacia xanthorrhiza* (Arracacha, Apiaceae)	It is found in South American Countries such as Ecuador, Colombia, Brazil and Venezuela [160].	Adapted to mesothermic, montane, day length regimes and tropical frost-free conditions [160].
42	*Morinda citrifolia* (Indian mulberry, Rubiaceae)	Native to Southeast Asia and Australia and widely distributed globally [161].	Vitamins such as ascorbic acid and provitamin A, amino acids such as aspartic acid, mineral and an alkaloid, xeronine, are detected in its fruits [162]. The plant shows tolerance to a number of stresses such as drought, water logging and salinity [161].
43	*Canavalia gladiata* (Sword bean, Leguminosae)	They are cultivated on a limited scale in Asia, West Indies, Africa and South America [163].	Seed coat of the sword bean is rich in gallic acid and other derivatives [164]. Seeds are a rich source of sodium, potassium and calcium [165]. The crude protein content of sword beans is high. Some cultivars are fairly resistant to drought [163].
44	*Lupinus mutabilis* (Tarwi, Leguminosae)	Distributed widely in the Andes, Venezuela, Colombia, Ecuador, Peru and Bolivia, Australia, Germany, New Zealand, Poland and the United Kingdom [166].	Seeds have high protein and lipid content whereas fiber and carbohydrate content are lower compared to other lupin species [167]. It has adaptability to temperate and cold climates. It can grow on marginal land and low fertility soils [168].
45	*Limonia acidissima* (Wood Apple, Rutaceae)	Native to India but also cultivated in Bangladesh, Pakistan and Sri Lanka [169].	The fruits are rich in β-carotene, vitamin B, vitamin C, thiamine and riboflavin. Fruit pulp is enriched with citric acid, other fruit acids, mucilage and minerals. Other compounds such as alkaloids, coumarins, fatty acids and sterols are also detected in its fruits [169]. It is well adapted to drier conditions and thus shows a greater stress tolerance [170].
46	*Cordia myxa* (Indian Cherry, Boraginaceae)	It is found globally especially in the tropics. It grows naturally in India, Myanmar and Afghanistan [171].	It displays drought tolerance and because of that it can easily grow in arid and semi-arid regions [171].
47	*Carissa carandas* (Karonda, Apocynaceae)	The plant is distributed in various parts of the world such as Nepal, Afghanistan, India, Sri Lanka, Java, Malaysia, Myanmar, Pakistan, Australia and South Africa [172].	Fruits are rich in calcium, iron, vitamin C, vitamin A [173]. The plant shows drought tolerance [172].
48	*Lepidium meyenii* (Maca, Brassicaceae)	Nutritionally highly valuable and is native to Peru [174].	It contains good quantities of fiber, essential amino acids, fatty acids, vitamin C and minerals such as copper, iron and calcium [175].
49	*Pastinaca sativa* (Parsnips, Apiaceae)	It is commonly found in old fields, roadsides and woodland edges in North America [176].	Rich in vitamins and minerals; particularly rich in potassium [176]. It shows drought tolerance [177].
50	*Xanthosoma sagittifolium* (American taro, Araceae)	Traditionally used as a tuber crop, native to Nigeria and tropical Africa [178].	Good source of carbohydrates and starch. Superior in terms of their protein digestibility and mineral composition such as calcium, phosphorus and magnesium [178].
51	*Colocasia antiquorum* (Taro, Araceae)	Widely consumed throughout the world especially Africa, Asia, the West Indies and South America [179].	The corms are full of anthocyanins [179]. They are salt tolerant [180].
52	*Nelumbo nucifera* (Lotus, Nymphaeaceae)	Creeping rhizomes are found throughout India; also found in China and Japan [181].	It is a good source of protein and total carbohydrates and possesses high calorific value. It also contains higher quantities of essential minerals such as Na, K, Mg, Fe, Co, Zn and P [182]. Exhibits flooding tolerance [183].
53	*Plectranthus rotundifolius* (Spreng, Lamiaceae)	Eaten for its edible tubers, native to tropical Africa. Grown in Africa and South East Asia [184].	It contains higher mineral content than potato, sweet potato and cassava [185]. Highly tolerant to drought [186].
54	*Triticum monococcum* (Einkorn wheat, Poaceae)	It has been an ancient staple food crop for many years. However, it is presently cultivated only in the Mediterranean region and continental Europe [187].	Not very good in dietary fiber but it contains good amounts of proteins, unsaturated fatty acids, zinc and iron. It contains antioxidant compounds such as carotenoids, tocols and conjugated polyphenols [187]. They exhibit tolerance to salinity and frost [188].
55	*Triticum dicoccon* (Emmer wheat, Poaceae)	Used as a cereal crop in the Middle- East, Central and West Asia and Europe [189].	Rich in proteins, carbohydrates and minerals, poor in fats [189]. Shows drought tolerance [190].
56	*Triticum spelta* (Dinkel wheat, Poaceae)	It has been an important staple food in parts of Europe in the ancient past [191].	High vitamin content [191] and rich source of iron, zinc, copper, magnesium, potassium, sodium and selenium [192]. They have high flooding tolerance [193].
57	*Eleusine coracana* (Finger millet, Poaceae)	It is produced in India, Niger, Mali, Burkina Faso, Chad and China [194].	It is rich in calcium, dietary fiber, protein, minerals, phenolics and vitamins such as thiamine and riboflavin. It contains a good quantity of iron and amino acids such as methionine, isoleucine, leucine and phenylalanine [194]. They are tolerant to drought, pests and pathogens [195].
58	*Panicum sumatrense* (Little millet, Poaceae)	Found in the Caucasus, China, India and Malaysia [196].	Rich in micronutrients such as calcium and iron. They also contain high dietary fiber content and essential amino acids and have low glycemic index [196]. It also shows considerable tolerance against drought, salinity stresses and diseases.
59	*Panicum miliaceum* (Proso millet, Poaceae)	Produced in China, Russia, India and some countries of Eastern Europe and North America [197].	The protein contains essential amino acids such as leucine, isoleucine and methionine than wheat [197]. They are drought tolerant [198].
60	*Pennisetum glaucum* (Pearl millet, Poaceae)	An important cereal in arid and semiarid regions of Asia and Africa [199].	It has high levels of calcium, iron, zinc, lipids and amino acids. Contains omega-9, omega-6 and omega-3 fatty acids. The tannins and phytates act as strong antioxidants [200,201]. It has a low glycemic index and it is a gluten-free crop. They are extremely drought-tolerant [202].
61	*Brosimum alicastrum* (Breadnut, Moraceae)	Grown in southern Mexico [203].	The flour obtained from the seeds is characterized by high protein, dietary fiber and micronutrient content. They are drought tolerant [204].
62	*Artocarpus altilis* (Breadfruit, Moraceae)	It is an important food in the Pacific [205].	Rich in fiber, protein, magnesium, potassium, phosphorus, thiamine (B1) and niacin (B3). They have tolerance to salinity and can grow on coralline soils and atolls [206].
63	*Mucuna pruriens* (Velvet bean, Fabaceae)	Cultivated in Southeast Asian countries, including India and Sri Lanka, and Central South American countries as a legume for its seeds [207].	The seeds are rich in dietary fiber and proteins [207]. They grow well in less fertile soil and show adaptation to drought conditions and acidified soils [208].
64	*Pachira aquatica* (Malabar Chestnut, Bombacaceae)	Native to Southern Mexico, Guyana and Northeastern Brazil and introduced in other areas such as Guangdong, Southern Yunnan and Taiwan as a cultivated plant [209].	Seeds contain a high amount of lipids, proteins with high amounts of essential amino acids such as tryptophan, threonine and phenylalanine/tyrosine [210]. Seeds contain more phosphate, magnesium, zinc, iron and copper than some fruits and other starchy foods [209].
65	*Strychnos cocculoides* (Monkey orange, Loganiaceae)	The species is native to Botswana, Kenya, Namibia, South Africa, Tanzania, Uganda, Zambia and Zimbabwe [211].	Adapted to drought prone and semi-arid areas. The vitamin C content of the fruits varies from 34.2 mg/100 g to 88 mg/100 g. Considered an essential source of iron [212].

**Table 2:** Overview of use of omics tools to identify genes/proteins/ions regulating important traits in traditional food plants.

Sl. No.	Traditional Food Plant	Distribution	Important Nutritional and Stress Resilient Traits	Exceptionally Notable Character	Applications of Different Omics Technologies
1.	*Eleusine coracana* (L.) Gaertn. (Finger millets, Poaceae)	Majorly produced in Mali, Niger, India, Burkina Faso and China [194].	Tolerant to pathogens and pests. Drought resistant. Rich in minerals such as calcium and iron, vitamins, protein, dietary fiber and phenolics [194,195].	Minerals and micronutrients are superior to rice and wheat [268].	1. Using genomics tools, Nirgude et al. [269] reported higher expression of *opaque2* (regulate seed storage proteins), *calcium transporters* and *calmodulin* gene (calcium storage) and Kumar et al. [270] discussed allele mining strategies for *PiKh* and *Pi21* genes that show resistance against *Pyricularia oryzae* blast disease. 2. Using transcriptomics, expression of several genes such as calcium transporters (*CaX*, *CDPKs*, *CBPs*) are reported [271]. Several transcription factors such as *MYB*, *MYC*, *WRKY* and *ZFD* were detected during drought stress [195]. 3. Proteomics study led to the identification of a calcium-binding protein, calreticulin [272]. Anatala et al. [273] reported heat shock proteins (HSPs), storage proteins and late embryogenesis abundant (LEA) during drought stress.
2	*Setaria italica* (L.) P. Beauv. (Foxtail millet, Poaceae)	Majorly cultivated in Asian countries such as India and China [150].	Great drought tolerant potential and grows well in barren and arid land [150].	Rich in essential amino acids, vitamin B, protein and micro elements [274].	1. Lata et al. [275] and Shi et al. [276] reported *POD* precursors, *late embryogenesis abundant* (*LEAs*) and *aquaporins* for drought tolerance by using transcriptomics. *Phospholipid hydroperoxide glutathione peroxidase* (*PHGPX*), *ascorbate peroxidase* (*APX*) and *catalase 1* (*CAT1*) during salinity tolerance were reported using transcriptomics by Sreenivasulu et al. [277].
3.	*Moringa oleifera* Lam. (Drumstick, Moringaceae)	Distributed mainly in Middle Eastern, African and Asian countries [278].	It has high micronutrient and vitamin content. It also shows antioxidant and medicinal activities. They can withstand occasional waterlogged conditions and adapt to hot and semi-arid conditions [279]. They are tolerant to heat, cold, salinity, nutrient starvation, variable light conditions and water deficiency [280].	Rich in micronutrients and vitamin A [279].	1. *WRKY* transcription factors for various abiotic stress tolerance and copies of *Cys_2_His*_2_ zinc finger motifs (*C2H2*), *APETALA2*/*ethylene-responsive element-binding protein* (*AP2-EREBP*), *C3H* transcription factors for drought and cold resistance were reported [280]. High-throughput sequencing technology reported microRNAs related to biotic and abiot stress tolerance [281]. Nutritional component-related genes such as *Vacuolar iron transporters* (*VIT*), *calreticulin* for calcium storage, Zinc transporters, magnesium transporter and genes for vitamin C biosynthesis recognised [282]. 2. Flavonoid compounds and rutinoside sugar compounds were detected using metabolomics by Makita [283].
4.	*Chenopodium quinoa* Willd. (Quinoa, Amaranthaceae)	Cultivated as an important crop since ancient times in various parts of North-Altiplano, South and Central Chile [284].	Rich source of minerals such as magnesium, iron, calcium, copper, potassium, zinc and phosphorus [66]. They have antioxidant activity (e.g., polyphenols) and rich in vitamins such as Vit. A, B1, B2, B9, C and E, lipids, proteins rich in essential amino acids particularly methionine and lysine, dietary fiber and carbohydrates [285]. They have extreme agro-ecological adaptability [286].	Higher mineral content than maize and barley including calcium, magnesium, iron, copper, potassium, phosphorus and zinc [66].	1. Draft gene sequence and genes related to abiotic stress and nutrients were identified [287]. 2. *Xyloglucan endotransglucosylase* genes, an *expansion A7-like* gene and *Ethylene Responsive Factor* (*ERF*) genes were found to be downregulated in salt-tolerant plants [288]. 3. Sobota et al. [289] reported albumin and globulins through proteomics. 4. Root cell membrane’s potential, net H^+^, Na^+^ and K^+^ fluxes during salinity adaptation through ionomics study [290].
5.	*Vigna unguiculata* (L.) Walp. (Cow pea, Fabaceae)	Cultivated across Africa, Southeast Asia, Latin Southern and the United States of America. It is not widely cultivated in Europe but used in some Mediterranean countries [291].	Rich in proteins and carbohydrates [292]. Proteins are rich in lysine and tryptophan amino acids [293]. Shows considerable adaptation to the warm climate with adequate rainfall [292].	High quantity of folic acid and low quantity of antinutrients [294].	1. Up-regulated expression of *chalcone isomerase* and *chalcone synthase* in the salt-tolerant plants were reported [295]. 2. Sugars, proline, galactinol and quercetin were identified as osmolytes during osmotic stress using metabolomics [296]. 3. Identified amino acids which are related to glycolysis and tricarboxylic acid cycle [297]. 4. Lutein and beta carotene were reported using metabolomics [298].
6.	*Vigna radiata* (L.) R. Wilczek (Mungbean, Leguminosae)	African regions, South and Southeast Asia [299].	Drought resistant. Higher iron and folate content [299].	Rich in digestible protein quantity than other pulses [300].	1. Eight flavonoids (vitexin, isovitexin, rutin, kaempferol 3-O-rutinoside, isoquercitrin, genistein, daidzein and isorhamnetin) and two phenolics were reported using metabolomics [299].
7.	*Sorghum bicolor* (L.) Moench(Sorghum, Poaceae)	Major food in semi-arid tropical temperatures of African and Asian regions [301].	Suitable for cultivation in dry areas and poor soil conditions [302]. Gluten-free cereal that is rich in antioxidants and phenolic compounds [303].	Gluten-free grains [303,304].	1. Quantitative trait loci for sorghum polyphenols were recognized [302]. 2. Increased expression of *Late Embryogenesis Abundant* (*LEA*), *delta 1-pyrroline-5-carboxylate synthase 2* (*P5CS2*) and high-affinity *K+ transporter 1* (*HKT1*) for drought tolerance [305]. Salinity and osmotic stress tolerance genes reported [306]. 3. Presence of fructose, galactose, lactose, cellobiose and sedoheptulose as an osmotic protectant were detected using metabolomics [307]. 4. Glutathione-S transferases and l-ascorbate peroxidase during salinity stress identified [308].
8.	*Manihot esculenta* Crantz. (Cassava, Euphorbiaceae)	Used by different communities all over the world, mainly tropical and subtropical areas [309].	Adapted to marginal soil conditions and erratic rain. Carbohydrate and protein rich [310].	Rich source of energy [311].	1. Using genomics, carotenoid markers on chromosome 1 and candidate genes for carotenoid (*phytoene synthase*) and starch biosynthesis were reported [312]. 2. Identification of starch biosynthesis genes [310]. Expression profiling and characterization of drought responsive *Abscisic acid* (*ABA*)*-responsive element* (*ABRE*)*-binding factors* (*ABFs*) [313]. Upregulation of 1300 genes during drought stress [314]. Transcription factors related to heat stress (*A3*, *heat-shock transcription factor 21* and a *homeobox-leucine zipper protein ATHB12*) and *dehydration tolerance* (*ERD1*, *RD19*, *RD22* precursor, *drought-induced protein Di19-like*) were reported [315]. *WRKY* genes related to abiotic stress tolerance [316]. 3. Proteomics—ATP synthase subunit beta, Rubisco activase (RCA), Rubisco, phosphoglycerate, chaperone peroxiredoxin, heat shock protein, glutathione transferase profiling during cold stress [317].
9.	*Amaranthus hypochondriacus* L., *Amaranthus viridis* L. (Amaranth, Amaranthaceae)	Consumed in China since ancient times. Central America, South America. It is also used in Africa and Caribbean [318].	Leaves and seeds are rich in quality proteins and its quantity is higher than maize. Proteins contain higher amounts of amino acid lysine and sulfur containing amino acids [319]. Amaranth oil contains unsaturated linolenic fatty acid which is good for human health [320].	High quality protein with rich lysine content in leaves and seed [319].	1. Gene annotation of lysine biosynthetic pathway and expression analysis was analyzed [321]. 2. Chloroplast chaperones, Rubisco large subunit, cytochrome b6f, oxygen evolving complexes and ascorbate peroxidase expression variation during drought stress were studied [322]. 3. Lutein and beta carotene detection [298].
10.	*Sesuvium portulacastrum* (L.) L. (Shoreline purslane, Aizoaceae)	Locally consumed in various regions of India, South East Asia, Philippines [323].	Salt, drought and oxidative stress tolerance. Salty taste and fleshy nature of leaves [324].	Rich source of sodium [323].	1. Identified *Late embryogenesis abundant 2* as the gene for salt and drought tolerance [324]. *Fructose-1,6-bisphosphate aldolase* gene (*FBA*) for abiotic stress tolerance was isolated [325]. 2. Copper (Cu), iron (Fe), manganese (Mn) and zinc (Zn) accumulation during salinity tolerance was reported [326].
11.	*Ipomoea batatas* (L.) Lam. (Sweet potato, Convolvulaceae)	Consumed throughout the world. Asia and Pacific islands produce 92 % of the world’s sweet potato supply [327].	It is pest and disease tolerant and adapted to high moisture conditions. Rich in complex carbohydrates, vitamin A, vitamin C, Fe and K. Orange-fleshed sweet potatoes are one of the storehouses of beta-carotene. It is a highly resistant crop [327].	Rich source of beta carotene [327].	*1. APX*, *manganese-dependent superoxide dismutase* (*MnSOD*), *LEA*, *early responsive to dehydration* (*ERD*), *sodium/hydrogen antiporter* (*NHX*), *aquaporin* (*AQP*), *vacuolar calcium ion transporter* (*CAX*), *metallothionein* (*MT*), *betaine aldehyde dehydrogenase* (*BADH*), *pyrophosphatase* (*PPase*), *catalase* (*CAT*), *polyphenol oxidases* (*PPO*), *ABRE-binding protein* (*AREB*) during abiotic stress tolerance reported [328]. 2. Amino acids, carbohydrates and flavonoids were detected using metabolomics [329]. Βeta-carotene content [330].
12.	*Ipomoea imperati* (Vahl) Griseb. (Beach morning glory, Convolvulaceae)	Distributed in coastline all over the world [331]. Consumed by local communities for the underground tuber.	Salinity tolerant and grows well in poor nutrient soil [331].	Rich in sodium [331].	1. Expression profiling of *AP2/EREBP*, *bHLH*, *HD-ZIP* and *MYB* transcription factors during salinity tolerance reported [331].
13.	*Dioscorea* spp. (Yam, Dioscoreaceae)	Tropical and subtropical Countries. Major food in Africa [309].	Great source of fiber, potassium, manganese, copper and antioxidants. They also exhibit abiotic stress tolerance [332].	Vitamin C and potassium rich [333].	1. Metabolite profiling revealed amino acid content, malic acid content, fatty acids and phosphate content [332]. 2. Genome sequencing revealed the hybrid origin of *Dioscorea rotundata* from *D. prehensilis* (wild rainforest plant) and *Dioscorea abyssinica* (Savannah adapted plant) [334].
14.	*Portulaca oleracea* L. (Common purslane, Portulacaceae)	Distributed all around the world such as New Zealand, Canada, America, temperate countries of Europe, Australia and is highly abundant in India [335].	It contains high amounts of α-linolenic acid and oxalic acid in their leaves which are highly health beneficial [336]. It is also rich in carbohydrates, protein, minerals (calcium, magnesium, sodium and potassium), vitamin C, carotene, riboflavin, thiamine and nicotinic acid. It is well adapted to dry and salinity conditions, therefore ideal for arid areas [337].	High amount of alpha-linolenic acid and oxalic acid in the leaves [336].	Metabolomics study reported 6 amino acids, 22 phenolic compounds, 16 alkaloids and 11 fatty acids [338]. α-linolenic acid accounted for about 40 % to 60 % of total fatty acid [339].
15.	*Physalis peruviana* L. (Wild tomatillos, Solanaceae)	A cultural staple of Mexico, Central America, South Africa, North America and Europe [340].	They have carotenoids, minerals and vitamin-rich fruits and seeds and show adaptability towards various environmental conditions [341,342].	Carotenoid and vitamin-rich fruits and seeds [341].	Metabolomic profiling reported lutein as the most abundant carotenoid (64.61 µg/g at the half-ripe stage) and the presence of gamma carotenoid (rare in fruits) [343].
16.	*Rumex vesicarius* L. (Ruby dock, Polygonaceae)	Cultivated in North Indian states as a vegetable [344].	Rich in phenols, ascorbic acid, α-tocopherol and β-carotene [345].	Vitamin rich [345].	Metabolomic study reported 13 Phenolic compounds, ascorbic acid, α-tocopherol and β-carotene content and 6-C-glucosyl-naringenin identified as the key phenolic compound which have high antioxidant capacity [345].
17.	*Corylus avellana* L. (Hazelnuts, Betulaceae)	Consumed by human civilizations from Mesolithic time onwards and cultivated worldwide especially in Spain, Turkey and Italy, United States and Canada [346,347].	Rich source of starch, protein, lipids, vitamin E and C, potassium, phosphorus, magnesium and calcium [348].	Rich in malic acid and unsaturated fatty acids [349].	Reported higher concentration of palmitic acid which prevents metabolic syndromes such as diabetes [350].
18.	*Avena sativa* L. (Oats, Poaceae)	Consumed in developing as well as developed countries [351].	Nutritionally rich, traditionally used cereal crops as a major protein diet in cold climate countries including Northern Europe [352]. Better adapted to acid soils and variable soil types than other grain cereal crops [351].	High dietary fiber content and 78–81.5% unsaturated fatty acids out of 5–9 % lipids [353].	1. Barley yellow dwarf virus tolerance QTL on chromosome 3C using genome wide association study was reported [354]. 2. Presence of polyamines detected during osmotic stress detected [355].
19.	*Bacopa monnieri* (L.) Pennell. (Brahmi, Plantaginaceae)	Sri Lanka, India, Nepal, China, Taiwan, Vietnam and Pakistan. Traditionally used as a medicinal plant from ancient times onwards [356].	Rich in Fe, Mg and Zn. Studies have proven the ability of Brahmi to enhance memory. They grow well in Marshy areas [356].	Rich source of microelements [356].	1. *De novo* assembly of transcriptome and draft chloroplast genome from RNAseq data [357]. 2. Proline content elevation during osmotic stress [358].
20	*Elaeagnus umbellata* Thunb. (Autumn olive, Elaeagnaceae)	Berries consumed in tropical and temperate Asia. Nowadays it is available in European countries also [65].	The berries are a rich source of lycopene and possess 10 times higher quantity of lycopene in their fruits than tomatoes [359]. They are rich in β-cryptoxanthin, α-cryptoxanthin, lutein, β-carotene, phytofluene and phytoene and vitamins. Exhibit drought tolerance, temperature tolerance and high tolerance to pruning. Can grow in high-saline soils [65].	Ten times higher quantity of lycopene in their fruit than tomato [65].	1. *Phytoene Synthase* (*EutPSY*) gene expression correlation with lycopene [360]. 2. Sugar metabolism-related enzymes (R-amylase, UGPase, phosphoglucomutase, acid invertase and triose-phosphate isomerase) and carotenoid biosynthesis-related proteins (Acetyl-CoA C-acetyltransferase, IPP isomerase and dimethylallyl diphosphate) reported [361].
21.	*Porteresia coarctata* (Roxb.) Tateoka(Wild rice, Poaceae)	India, Sri Lanka, Bangladesh and Myanmar [362].	Grows in saline estuaries and is adapted to salinity [362].	With increase in salinity stress, carbohydrate and ash content increases [363].	Elevation of proteins related to photosynthesis such as Rubisco large subunit, Rubisco small subunit and light harvesting complex-chlorophyll a, b reported during salinity [362].
22.	*Atriplex lentiformis* (Torr.) S.Watson(Quail Bush, Chenopodiaceae)	South western United States and northern Mexico [290].	Good salinity adaptation capacity [290].	Rich source of sodium [364].	1. Studied the H+-ATPase activity of plasma membranes during salinity stress, which leads the plant for K+ retention and Na+ exclusion for better salt tolerance [290].
23	*Fagopyrum esculentum* Moench (Buckwheat, Polygonaceae)	Worldwide distribution [365].	Grows in hilly areas and marginal ecosystems [365]. Rich in sulfur containing amino acids such as cysteine and methionine than any cereal. Fatless, gluten-free grains that are rich in starch and minerals such as Ca, Mo, S and vitamins [352,366].	Excellent quality of protein with a high amount of essential amino acid lysine [98].	1. Draft genome of buckwheat was developed and the same study identified expression of three granule bound *starch synthase* (*GBSS*) genes [287]. 2. Differential expression of sugar biosynthesis and metabolism-related genes in *F. esculentum* and *F. tataricum* [367].
24	*Panicum miliaceum* L. (Proso millet, Poaceae)	It is cultivated widely in Asian countries, some African countries and the Middle East [368].	More efficient in water usage, because it shows the C4 pathway, hence suitable for cultivation in dry areas. High productivity in low input soil and marginal lands [263]. Rich in both major nutrients and minor nutrients such as phenolics, minerals and vitamins. Gluten-free grain [197].	Richer in essential amino acids than wheat [197].	1. Genes related to C4 mechanisms such as *carbonic anhydrase* (*CA*), *NAD dependent malic enzyme* (*NAD-ME*) and *NADP- malic enzyme* (NADP-ME) [369]. 2. Protein related to metabolisms such as polysaccharide and starch [370]. 3. Nearly 48 metabolites including several primary metabolites and five phenolic acids were detected [371].
25	*Sclerocarya birrea* (A.Rich.) Hochst. (Marula, Anacardiaceae)	Popular African tree [140].	Ascorbic acid-, arginine- and glutamine-rich fruits [140].	Highest level of arginine and ascorbic acid [140].	1. Draft genome reported and identified genes involved in starch biosynthesis pathway [265].
26	*Ziziphus jujuba* Mill. (Chinese jujube, Rhamnaceae)	Mainly cultivated in Asian countries [372].	Salt tolerant and drought tolerant [373]. Good source of phenolics, vitamin C, triterpenic acids, flavonoids and polysaccharides [374].	Rich in unsaturated fatty acid, especially omega-6 fatty acid [375].	1. Expression of 5269 differentially expressed genes during salinity were recognized and among them, 2540 were downregulated and 2729 were upregulated [373]. Expression profiling of genes during heat stress led to identification of heat responsive factors [374].Expression profiling of three *UDP-glucose flavonoid 3-O-glucosyltransferase* (*UFGT*), responsible for anthocyanin accumulation in fruit peel [376].
27	*Dacryodes edulis* (G.Don.) H.J.Lam (African pear, bush pear, Burseraceae)	Cultivated in tropical countries of Africa [113].	Rich source of protein, vitamins and lipids [113].	Selenium content is high compared to other crops reported with selenium. Beta-carotene is higher than papaya, avocado and amaranth. They are rich in potassium [114].	NA
28	*Basella alba* L. (Vine spinach, Basellaceae)	Tropical Asian countries [89].	Heat- and drought-tolerant plants, high quantities of vitamin A, C, iron and calcium are present [89].	Leaves are rich in calcium [89].	NA
29	*Solanum quitoense* Lam. (Lulo, Solanaceae)	South American countries and nowadays found in European countries also [377].	Adapted to shady areas and rich in vitamins [377].	Rich in carotenoids [143].	NA
30	*Chenopodium pallidicaule* Aellen (Cañiwa, Amaranthaceae)	Mainly cultivated in Bolivia and Peru [378].	Disease and pest resistant. Adapted to salinity, heat and drought tolerance. Rich in protein [378].	Exceptional protein content and quality, equivalent to that of milk proteins. Balanced amino acid composition [135].	NA
31	*Adansonia digitata* L. (Baobab, Malvaceae)	Tropical African countries [120].	Adapted to arid and semi-arid conditions and rich source of vitamin A and C [120].	Fruit pulp vitamin C is almost ten times that of oranges [121].	Performed profiling of proteins, amino acids and minerals [121].
32	*Strychnos cocculoides* Baker (Monkey orange, Loganiaceae)	America, African and South tropic Asian regions [379].	Adapted to warm climate conditions [379]. Rich in iron, zinc and vitamin C [212].	Essential source of iron [212].	N/A
33	*Panicum sumatrense* Roth(Little millet, Poaceae)	Tropical region of Asia and Africa [368].	Grow with minimal requirements and adapted to harsh environmental conditions and rich in micronutrients [368].	Grains are a good source of iron and calcium [196].	1. Complete chloroplast genome was sequenced [380]. 2. RNa sequences were performed and differential gene expression at the time of drought and salinity stress also studied. At the time of drought stress, 241 DGEs were upregulated and 134 DGEs were downregulated [381].

**Table 3:** Genes governing major domestication traits in crop plants related to traditional food plants [437].

Domesticated Crop|Related Traditional Plant (S)	Gene	Wild Trait	Domestication Trait	Function of the Gene	Reference (s)
*Fragaria vesca*|*Pyrus pyrifolia, Rubus fruticosus, R. spectabilis, R. occidentalis* [459].	*TERMINAL FLOWER 1 Homologue KSN* (*TFL1*)	Non-frequent flowering.	Continuous flowering.	Flowering repression. Establishment of a continuous flowering habit.	[437,460,461]
*Hordeum vulgare|H. murinum* [462], *H. brachyantherum*, *H. jubatum* [463].	*nud* (*nud*)	Palea and lemma hulls are tightly adhered to the caryopsis which results in hulled seeds.	Reduced organ adhesion between the caryopsis and the hull.	Controls caryopsis and is involved in the lipid biosynthesis pathway.	[437,464]
	*SIX-ROWED SPIKE1* (*VRS1*)	Two-rowed inflorescences.	Change in inflorescence architecture from two-rowed to six-rowed spikelet.	Loss of function of Vrs1 results in the conversion of the rudimentary lateral two-rowed spikelet in barley into a fully developed six-rowed fertile spikelet.	[437,465]
	*Photoperiod-H1* (*Ppd-H1*)	Early flowering.	Delayed flowering time.	Candidate gene for leaf size and flowering time in the barley population.	[437,466]
	*RESISTANT TO RALSTONIA SOLANACEARUM 2* (*RRS2*)	Low leaf scald resistance.	Increased leaf scald resistance.	Resistance gene to fungal pathogen *Rhynchosporium secalis* which causes leaf scald disease.	[437,467]
	*EARLY FLOWERING3* (*ELF3*)	Late flowering.	Earlier flowering time.	Part of a circadian clock input pathway. Can regulate the initiation of flowering independently of phyB.	[437,468]
	*INTERMEDIUM-C* (*INT-C*)	Tillering and sterile lateral spikelets.	Increased expression causes suppression of tillering and male fertility in lateral spikelets.	Regulation of shoot system development. Mutation of the gene is correlated with lateral spikelet fertility phenotypes.	[437,469]..
*Oryza sativa|O. latifolia*, *O. glumaepatula* [470].	*PROSTRATE GROWTH1* (*PROG1*)	Prostrate growth.	Asymmetrical growth of the tiller base leading to erect growth.	Inactive prog1 results in the conversion of prostrate to erect growth habit in domesticated rice.	[437,471]
	*SHATTERING4-1* (*SH4-1*)	Easily shatters seeds.	Lack of an abscission layer leads to seed non-shattering.	Responsible for rice grain shattering.	[437,472,473]
	*BLACK HULL4* (*BH4*)	Black hull.	White hull.	Controls black hull color.	[437,474]
	*Red pericarp* (*Rc*)	Red pericarp.	White pericarp (absence of anthocyanin).	Required for red pericarp in rice- proanthocyanin synthesis-related gene.	[437,475]
	*AMMONIUM TRANSPORTER1;1* (*AMT1;1*)	Poor nitrogen uptake mechanism.	Modified nitrogen uptake and response.	It is a high affinity ammonium transporter which may be involved in ammonium uptake from the soil.	[437,476]
	*LIGULELESS1* (*LG1*)	Open the panicle and easily shatter seeds.	Altered panicle growth results in closed panicles and reduced shattering.	Controls laminar joint formation between leaf blade and leaf sheath and controls ligule and auricle development.	[437,477]
	*BETAINE ALDEHYDE DEHYDROGENASE2* (*BADH2*)	Non-fragrant grains.	Fragrant grains.	Plays a key role in the accumulation of a fragrant compound, 2-acetyl-1-pyrroline (2AP). An inactive BADH2 promotes fragrance in rice.	[437,478]
	*GRAIN WIDTH5* (*GW5/SW5)*)	Small sized seeds.	Increase seed size by increasing the cell number of the outer glume layer.	Controls rice grain width and weight.	[437,479]
	*GRANULE BOUND STARCH SYNTHASE I* (*Waxy*; *GBSSI)*	Non-glutinous grains.	Glutinous grains.	It controls amylose synthesis in the endosperm.	[437,480,481]
	*GRAIN SIZE3* (*GS3*)	Short grain.	Long grain phenotype.	Contributes to seed or grain size.	[437,482]
	*SHATTERING1* (*Sh1*)	Shattering.	Reduction in shattering.	Controls shattering.	[437,472]
	*HEADINGDATE1* (*HD1*)	Early flowering.	Delayed flowering time.	A regulator of the florigen gene Hd3a.	[437,483]
	*Quantitative trait locus of seed shattering on chromosome 1 (qSH1)*	Shattering seeds.	Loss of seed shattering because of the absence of an abscission layer.	Regulates seed shattering.	[472,484]
*Zea mays|Setaria italica, Lolium perenne, Digitaria exilis, Avena sativa, Secale cereale* [485].	*teosinte glume architecture 1* (*Tga1*)	Hard glume.	Softer glume.	Represses branching.	[437,486,487,488,489]
	*zea agamous-like1* (*Zagl1*)	Small female ear.	Increase in female ear length.	Role in flowering time and ear size.	[437]
	*ramosa1* (*ra1*)	Many branches with multiple ears on each branch and tassel at the tip of the branch.	Affects kernel organization, altered inflorescence architecture.	Regulate the inflorescence branching systems.	[437,490]
	*PROLAMIN BINDING FACTOR* (*PBF*)	Less protein storage.	Altered prolamin protein levels in seeds.	Controls the expression of seed storage protein (zein) genes.	[437]
	*teosinte branched 1* (*TB1*)	Many branches with multiple ears on each branch and tassel at the tip of the branch.	Increased expression causes short, ear-tipped branches.	It is involved in apical dominance. It has a significant role in repression of axillary organs.	[437,487,489,491].
	*SHATTERING 1-5.1, SHATTERING1-5.2* (*Sh1-5.1-Sh1-5.2*)	Easily shattering.	Non-shattering phenotype because of lack of abscission layer.	It plays a key role in establishment of the abscission layer and is responsible for grain shattering.	[437,472]
	*BARREN STALK1* (*BA1*)	Presence of axillary meristem.	Prevents axillary meristem development.	Modulates maize inflorescence. Regulates vegetative lateral meristem.	[437,492]
	*CO, CO-LIKE and TIMING OF CAB1* (*CCT*)	Late flowering.	Lower expression leads to earlier flowering.	*CO*, *CO-like* and *TIMING OF CAB1* modulate flowering time.	[437,493,494]
	*MADS19* (*zmm19*)	Kernels without glume covering.	Ectopic expression in inflorescences leads to kernels covered by glumes.	Loss of the *MADS19* results in larger glumes.	[437,495]
	*SUGARY1* (*Su1*)	Non-sweet taste.	Altered starch biosynthesis, sugary sweet taste.	Key role in starch biosynthetic process	[437,496,497]
	*SHATTERING1* (*Sh1*)	Shattering phenotype.	Non-shattering phenotype because of lack of abscission layer.	Promotes grain shattering through an abscission layer.	[437,472]
*Glycine max|Canavalia ensiformis, C. gladiata, Lupinus mutabilis, Cajanus cajan, Phaseolus mungo, P. vulgaris, P. aconitifolius, P. calcaratus, P. lunatus, Vigna unguiculata, Lens culinaris, Vicia faba, Lathyrus sativus, Cyamopsis tetragonolobus, Dolichos lablab, Arachis hypogaea* [498,499].	*TERMINAL FLOWER1b* (*TFL1b*)	Indeterminate shoots.	Determinate shoots end with terminal inflorescence.	Maintains indeterminate growth of cells in the shoot apical meristem.	[437]
*Setaria italica|S. faberi, S. viridis, S. pumila, Panicum glaucum, P. miliaceum* [500].	*GRANULE BOUND STARCH SYNTHASE I* (*GBSSI*)	Non-glutinous grains.	Glutinous grains.	The gene is involved in starch biosynthesis.	[437,501,502]
*Solanum lycopersicum|S. quitoense, S. macrocarpon, Physalis prunisa, P. minima* [446,503].	*FASCIATED* (*FAS*)	Small fruit size.	Increased cell proliferation leads to larger fruit.	Promotes cell size growth.	[437,504]
	*fruit weight 2.2* (*FW2.2*)	Lower number of locules.	Increase in locule number in fruit.	Regulates fruit size.	[437,505,506]
	*OVATE* (*OVATE*)	Non-expansive fruit neck region.	Expansion of the fruit and fruit shape determination.	Key regulator of fruit shape.	[437,507]
	*SUN* (*SUN*)	Fruit is not elongated.	Increased growth resulting in elongated fruit.	Major gene controlling the elongated fruit shape.	[437,508]
	*LOCULE NUMBER* (*LC*)	Fruits have two locules.	Fruits have 3–4 locules instead of 2 locules.	Control fruit shape.	[437,504]
*Vitis vinifera*|*Cissus discolor, C. s mollissima, Cayratia pedata, Ampelocissus latifolia* [509].	*myb-related transcription factor* (*MYBA1*)	Dark colored berry.	Lack of anthocyanins lead to white berry color.	Controls the last steps in the anthocyanins biosynthesis pathway.	[437,510]
	*myb-related transcription factor* (*MYBA2*)	Dark colored berry.	Lack of anthocyanins lead to white berry color.	Control the anthocyanin biosynthesis pathway.	[437]

**Table 4:** Examples of gene editing in major and few minor crops.

Sl. No.	Crop Name	Method of Gene Editing	Target Gene and Effect of Mutation after Editing	References
1	*Solanum lycopersicum* L.	CRISPR/Cas9 system via *Agrobacterium-* mediated transformation and TALEN.	*Anthocyanin mutant 1* (*ANT1)*—resulted in deep purple colored plant tissues.	[526]
		CRISPR/Cas9 system via *Agrobacterium-* mediated transformation.	*Mildew resistance locus O* (*MLO*)—powdery mildew-resistant plant.	[527]
2	*Solanum tuberosum* L.	CRISPR/Cas9 system via *Agrobacterium-* mediated transformation.	*Acetolactate synthase1* (*ALS1*)—resulted in reduced herbicide susceptibility.	[528]
		CRISPR/Cas9 system PEG mediated protoplast transfection.	*Granule bound starch synthase* (*GBSS*)—resulted in absence of amylase enzyme.	[529]
3	*Zea mays* L.	CRISPR/Cas9 system via particle bombardment transformation.	*ALS1, ALS2*—resulted in chlorsulfuron-resistant plants.	[513]
		CRISPR/Cas9 system via particle bombardment transformation.	*Auxin-regulated gene involved in organ size* (*ARGOS8*)—resulted in decreased ethylene response and increased grain yield under stress conditions.	[530]
		CRISPR/Cas9 system via *Agrobacterium-* mediated transformation.	*Thermosensitive genic male-sterile 5* (*TMS5*)-resulted in male sterility.	[531]
		TALEN via PEG-mediated transformation.	*Phytoene desaturase* (*PDS*), *Inositol-pentakisphosphate 2-kinase* (*IPK1A*), *Isopentenyl phosphate kinase* (*IPK*), *Multidrug resistance-associated protein 4* (*MRP4*)—resulted in mutation of the genes.	[532]
		CRISPR/Cas9 system via PEG-mediated transformation.	*Inositol phosphate kinase* (*IPK*)—resulted in mutation.	[532]
		CRISPR/Cas9 system.	*G protein β subunit* (*Gβ*)—resulted in an autoimmune response.	[533]
		CRISPR/Cas9 system.	*Waxy*—resulted in waxy corn hybrids.	[534]
		CRISPR/Cas9 system via *Agrobacterium-* mediated transformation.	*Gibberellin-Oxidase20-3* (*GA20ox3*)—resulted in semi dwarf plants.	[535]
4	*Oryza sativa* L.	CRISPR/Cas9 system via particle bombardment transformation.	*ALS*—resulted in herbicide resistance.	[536]
		CRISPR/Cpf1 system via particle bombardment transformation.	*Chlorophyllide-a oxygenase* (*COA1*) -resulted in precise gene insertions and indel mutations.	[537]
		CRISPR/Cas9 system via particle bombardment transformation.	*Nitrate transporter 1.1* (*NRT1.1B*)—resulted in improved nitrogen use efficiency.	[538]
		CRISPR/Cas9 system via PEG mediated transformation.	*Drooping leaf* (*DL*)—resulted in a drooping leaf phenotype.	[539]
5	*Triticum aestivum* L.	CRISPR/Cas9 system via *Agrobacterium-* mediated transformation.	*Grain width* (*GASR7*)—resulted in mutations.	[531]
		CRISPR/Cas9 system via particle bombardment transformation.	*Grain weight* (*GW*)—resulted in mutation of the gene.	[540]
6	*Malus domestica* Borkh.	CRISPR/Cas9 system via PEG mediated transformation.	*DIPM-1, DIPM-2* and *DIPM-4*—resulted in mutation of the genes..	[516]
7	*Vitis vinifera* L.	CRISPR/Cas9 system via PEG mediated transformation.	(*MLO-7*)—Resulted inmutations of the gene.	[516]
8	*Brassica oleracea* L.	CRISPR/Cas9 system via PEG mediated transformation.	*FRIGIDA* (*FRI) and phytoene desaturase (PDS)*—resulted in the mutations of the genes.	[541]
9	*Cucumis sativus* L.	CRISPR/Cas9 system via *Agrobacterium-* mediated transformation.	*WPP domain-inter*acting protein 1 (*WIP1*)—resulted in development of gynoecious phenotype with upper node having only female flowers.	[542]
		CRISPR/Cas9 system via *Agrobacterium-* mediated transformation.	*Eukaryotic translation initiation factor 4E* (*eIF4E*)—resulted in resistance against vein yellowing virus (ipomovirus), Zucchini yellow mosaic virus and Papaya ringspot mosaic virus-W (potyviruses).	[515]
10	*Solanum nigrum* L.	CRISPR/Cas9 system via *Agrobacterium-*mediated transformation.	Gravity response gene (*Lazy1*)—resulted in downward growth of the stem.	[543]
11	*Brassica rapa* L.	CRISPR/Cas9 system via PEG mediated transformation.	*FRI* and *PDS* genes—resulted in the mutations of the genes.	[541]
12	*Musa* x *paradisiaca* L.	CRISPR/Cas9 system via PEG mediated transformation.	*PDS*—resulted in mutation of the gene.	[544]
13.	*Nicotiana tabacum* L.	CRISPR/Cas9 system.	*PDS*—resulted in albino phenotype.	[545]
14	*Setaria viridis* (L.) P. Beauv.	CRISPR/Cas9_Trex2 system via *Agrobacterium-*mediated transformation.	*Domains rearranged methylase* (*Drm1*) and *male sterile 45* (*Ms45*)— resulted in the mutations of the genes.	[546]
		CRISPR/Cas9 system.	*Less Shattering1* (*Les1*)—reduced shattering.	[547]
15	*Medicago truncatula* Gaertn.	CRISPR/Cas9 system via *Agrobacterium-* mediated transformation.	*Hua enhancer1* (*Hen1*)—results in a shrunken, shriveled seed phenotype.	[548]
		CRISPR/Cas9 system via *Agrobacterium-* mediated transformation.	*PDS*—resulted in albino phenotypes.	[549]
16	*Vigna unguiculata* (L.) Walp.	CRISPR/Cas9 system via *Agrobacterium-* mediated transformation.	*Meiosis gene* (*SPO11-1*)—infertile phenotype.	[550]
		CRISPR/Cas9 system via *Agrobacterium-* mediated transformation.	*Symbiosis receptor-like kinase* (*SYMRK*)—resulted in blockage of nodule formation.	[525]
17	*Cicer arietinum* L.	CRISPR/Cas9 system via PEG mediated transformation.	*4-coumarate ligase* (*4CL*) *and Reveille 7* (*RVE7*) *genes*—resulted in mutations of the genes.	[551]

## Abbreviations

AOCC	African Orphan Crops Consortium
Cas9	CRISPR-Associated Protein 9
CFF	Crops For the Future
CRISPR-Cas9	Clustered Regularly Interspaced Short Palindromic Repeat-Associated Protein 9
DGE	Differential Gene Expression
DNA	Deoxyribonucleic Acid
FAO	Food and Agricultural Organization
GC	Gas Chromatography
gRNA	Guide ribonucleic Acid
HDR	Homology Directed Recombination
HPLC	High Performance Liquid Chromatography
ICP-MS	Inductively Coupled Plasma Mass Spectroscopy
ICRAF	International Council for Research in Agroforestry
mRNA	Messenger Ribonucleic Acid
NCBI	National Center for Biotechnology Information
NHEJ	Non-Homologous End Joining
PEG	Poly Ethylene Glycol
sgRNA	Single Guide Ribonucleic Acid
RNA	Ribonucleic Acid
RT-PCR	Real-Time Polymerase Chain Reaction
QTLs	Quantitative Trait Locus
TALENs	Transcriptional Activator-Like Effector Nucleases
TFPs	Traditional Food Plants
Trex2	Three prime Repair Exonuclease 2
ZFNs	Zinc Finger Nucleases
